# Inactivation of the *SLC25A1* gene during embryogenesis induces a unique senescence program controlled by p53

**DOI:** 10.1038/s41418-024-01428-w

**Published:** 2024-12-29

**Authors:** Anna Kasprzyk-Pawelec, Mingjun Tan, Raneen Rahhal, Alec McIntosh, Harvey R. Fernandez, Rami M. Mosaoa, Lei Jiang, Gray W. Pearson, Eric Glasgow, Jerry Vockley, Christopher Albanese, Maria Laura Avantaggiati

**Affiliations:** 1 Georgetown University Medical Center, Lombardi Comprehensive Cancer Center, Washington, D.C., USA; 2 Penn State College of Medicine, Department of Cellular & Molecular Physiology, Hershey, PA USA; 3 University of Virginia Medical Center, Charlottesville, VA USA; 4 Department of Biochemistry, Faculty of Science, King Abdulaziz University, Jeddah, Saudi Arabia; 5 Department of Molecular and Cellular Endocrinology, Arthur Riggs Diabetes and Metabolism Research Institute, City of Hope Medical Center, Comprehensive Cancer Center, City of Hope Medical Center, Duarte, CA USA; 6 Department of Pediatrics, Division of Genetic and Genomic Medicine, UPMC Children’s Hospital of Pittsburgh, Pittsburgh, PA USA

**Keywords:** Metabolic disorders, Development

## Abstract

Germline inactivating mutations of the *SLC25A1* gene contribute to various human disorders, including Velocardiofacial (VCFS), DiGeorge (DGS) syndromes and combined D/L-2-hydroxyglutaric aciduria (D/L-2HGA), a severe systemic disease characterized by the accumulation of 2-hydroxyglutaric acid (2HG). The mechanisms by which *SLC25A1* loss leads to these syndromes remain largely unclear. Here, we describe a mouse model of *SLC25A1* deficiency that mimics human VCFS/DGS and D/L-2HGA. Surprisingly, inactivation of both *Slc25a1* alleles results in alterations in the development of multiple organs, and in a severe proliferation defect by activating two senescence programs, oncogene-induced senescence (OIS) and mitochondrial dysfunction-induced senescence (MiDAS), which converge upon the induction of the p53 tumor suppressor. Mechanistically, cells and tissues with dysfunctional SLC25A1 protein undergo metabolic and transcriptional rewiring leading to the accumulation of 2HG *via* a non-canonical pathway and to the depletion of nicotinamide adenine dinucleotide, NAD^+^, which trigger senescence. Replenishing the pool of NAD^+^ or promoting the clearance of 2HG rescues the proliferation defect of cells with dysfunctional SLC25A1 in a cooperative fashion. Further, removal of p53 activity *via* RNA interference restores proliferation, indicating that p53 acts as a critical barrier to the expansion of cells lacking functional SLC25A1. These findings reveal unexpected pathogenic roles of senescence and of p53 in D/L-2HGA and identify potential therapeutic strategies to correct salient molecular alterations driving this disease.

## Introduction

The solute carrier family member SLC25A1, also known as CTP/CIC, belongs to a family of nuclear-encoded ion transporters localized in the inner mitochondrial membrane where it promotes the bi-directional exchange of citrate between the mitochondria and the cytosol [1–3]. In the cytoplasm, citrate is a substrate for de novo lipid synthesis (DNL), while in the mitochondria it feeds into the tricarboxylic acid (TCA) cycle. The human *SLC25A1* gene maps to chromosome *22q11.2*. Heterozygous microdeletions of this region give rise to Velocardiofacial (VCFS)/ DiGeorge (DGS) syndromes [4, 5], a group of developmental disorders associated with loss of 30 to 40 genes in addition to *SLC25A1*. Patients with *22q11.2* deletion syndrome present with heart malformations, cleft palate, immune deficiency, thymic aplasia, short stature, and abnormal facial features, particularly microcephaly, abnormal ears and eyes, and underdeveloped chin. We and others have previously shown that the knock-down of the *slc25a1a* homologous gene in zebrafish embryos induces craniofacial and heart abnormalities, suggesting a role for SLC25A1 in these human disorders [6, 7].

Various *SLC25A1* gene mutations have also been reported in a group of developmental syndromes named D/L-hydroxyglutaric aciduria (D/L-2HGA), hallmarked by the accumulation and urinary excretion of the D- and L- enantiomers of 2-hydroxyglutarate (2HG) [8–13]. The clinical spectrum of manifestations associated with *SLC25A1* gene mutations in D/L-2HGA is severe, consisting of craniofacial abnormalities, facial dysmorphic features, micrognathia or retrognathia, and microcephaly or macrocephaly [13]. In addition, these patients present with developmental delay, brain abnormalities, (cerebral atrophy, agenesis of the corpus callosum), and respiratory insufficiency, epilepsy, encephalopathy and occasionally, cardiomyopathy [8]. Unlike in VCFS/DGS, where only one allele of the *SLC25A1* gene is lost, D/L-2HG-aciduria results from homozygous or compound heterozygous mutations that typically involve a profound disruption of the citrate transport activity. The most severe cases of D/L-2HGA are sustained by the compound heterozygous alleles, p.Ala9Profs*82 and p.Pro45Leu (onset at 1 day, death at 1 month) [11, 13], which result in complete loss of SLC25A1 mitochondrial activities. In all other cases, with few exceptions, there is a strong correlation between the extent of loss of export activity and the severity of the clinical manifestations.

**Fig. 1:**
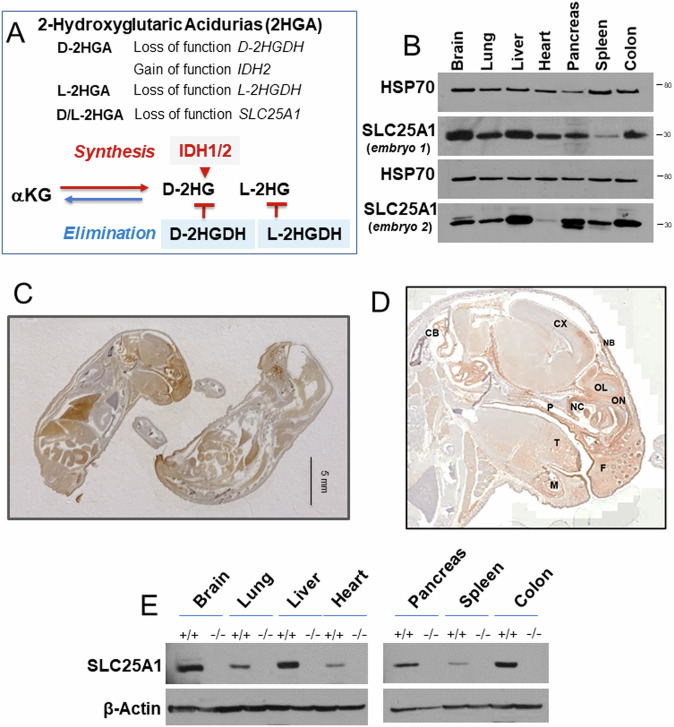
Analysis of SLC25A1 protein expression. **A** Schematic representation of 2-Hydroxyglutaric acidurias (2HGA). Upper panel: genetic alterations in individual and combined 2-HGA. Lowe panel: some of the main pathways to the synthesis and elimination of D- and L-2HG in 2HGA. **B** Expression levels of SLC25A1 protein in the indicated organs derived from two *Slc25a1* wild-type embryos at E19.5 dpf. **C**, **D** Representative IHC images with the anti-SLC25A1 antibody staining in embryos at E19 dpf. Cb= Cerebellum, Cx = Cerebral cortex, F=follicle, P=palate, T = tongue, M = mandible, NB= nasal bone, NC = nasal cavity, OL=olfactory lobe, ON=olfactory nerve. **E** Immunoblot with the anti-SLC25A1 antibody in the indicated organs derived from wild-type or SLC25A1 deficient embryos at day 19.5 pdf.

It is still unknown whether 2HG plays a direct role in the pathogenesis of SLC25A1-associated D/L-2HGA. Both enantiomers are derived from the reduction of 2-oxoglutarate (α-ketoglutarate, αKG) and there are different pathways for 2HG production and elimination (Fig. 1A) [14–16]. Individual forms of L- or D-2HGA are caused by mutations in the 2-hydroxyglutarate dehydrogenases L- (*L2HGDH*), *or D*- (*D2HGDH*), which eliminate each enantiomer by oxidation to αKG. Gain of function, mutant forms of isocitrate dehydrogenase 1 or 2 (*IDH1*/2) convert αKG to D-2HG, and *IDH2* mutations can cause individual D-2HGA [8, 14]. Mechanistically, D/L-2HG have been extensively characterized in the context of tumors harboring mutations of *IDH1/2* [16], which produce extraordinarily high (10–30 mM) amounts of these metabolites. By contrast, the levels of D-2HG and L-2HG in patients affected by D/L-2HG sustained by SLC25A1 deficiency, are only moderately elevated [16]. Furthermore, distinct biochemical alterations separate D/L-2HGA sustained by *SLC25A1* deficiency from other forms of 2HGA [11, 12, 17]. Indeed, we have documented that inhibition of SLC25A1 in tumor cells impairs OXPHOS, albeit with yet unknown molecular mechanisms [3, 18].

Senescence can be induced by various stress signals, including telomere attrition, oxidative stress, and genomic instability, and recruits the activity of tumor suppressors, including p53, pRb and p16/INK4a, to elicit arrest of the cycle. Senescent cells secrete many factors known as the Senescence-Associated Secretory Phenotype (SASP), which differ depending upon the upstream triggering signal(s). Oncogene induced senescence (OIS), is characterized by the secretion of pro-inflammatory mediators IL-6, IL-1β, and IL-1α, and involves the activity of several downstream effectors, including NFkB, mTOR, AP1/MAPK signaling, and the JAK/STAT pathway [19–22]. Due to DNA replication stress imposed by oncogenes, OIS is often associated with the DNA damage response, hallmarked by induction of phosphorylated histone gamma H2AX and of the kinase Chk1/2 [23]. A second form of senescence, mitochondrial dysfunction-induced senescence (MiDAS) contributes to accelerated aging in murine models of progeroid syndromes [24], and instead lacks pro-inflammatory cytokines while recruiting the activity of AMPk, TNFα, and of anti-inflammatory IL-10, among others [24–26]. MiDAS occurs when the NAD + /NADH ratio drops, leading to mitochondrial dysfunction and activation of the hypoxia-inducible factor, HIF1α in normoxic conditions, defined as a pseudo-hypoxia response. Importantly, both OIS and MiDAS activate p53 to enforce growth arrest.

In the present work we show that mice lacking both *Slc25a1* alleles exhibit several phenotypic characteristics typical of VCFS/DGS as well as of D/L-2HGA. Mechanistically, loss of SLC25A1 activity leads to a severe deficit of the proliferative capacity and uniquely triggers two distinct pathways of cellular senescence, MiDAS, and OIS. We identify several potential corrective strategies to reverse the molecular alterations and these senescence programs, potentially opening novel therapeutic interventions in disorders sustained by SLC25A1 deficiency.

## Methods

*Additional methods are included in the Supplemental file*

### Cell culture

The A549 and H1299 cell line were obtained from the tissue culture core facility at LCCC. Cells were grown in Dulbecco’s Modified Eagle’s Medium (DMEM 4500 mg/L glucose) supplemented with 10% fetal bovine serum, 4 mM L-glutamine, 1 mM sodium pyruvate, and 1% antibiotic solution. The S*LC25A1*-specific shRNA vectors were purchased from Sigma (TRCN0000232825; TRCN0000255350) and validated before (6,18,27). Octyl-2-HG (Cayman 16366, 16367) was diluted in PBS pH 7.2. NMN was purchased form Selleckchem (S5259) and DCA from Sigma (347795).

### Mice

The S*lc25a1* heterozygous mice were purchased from the Mutant Mouse Resource Research Center (MMRRC) (C57BL/6N-*Slc25a1*^*tm1a(EUCOMM)Wtsi*^, RRID:MMRRC_042258-UCD). The targeting vector allows for constitutive or conditional deletion of the *SLC25A1* gene through the incorporation of an *IRES:lacZ* trapping cassette which is inserted between introns 1 and 5 of the *Slc25a1* gene [27]. Mice were maintained on a 12 h light/dark cycle and were supplied with food and water ad libitum. To obtain mouse lacking both *Slc25a1* alleles, we crossed heterozygous *Slc25a1*^+/−^ mice. Pregnancy was confirmed by checking for vaginal plugs and the increase in body weight measured weekly.

## Results

### Loss of the *Slc25a1* gene during embryogenesis disrupts multiple organs development

To understand how SLC25A1 impinges upon embryonic development, we first studied the pattern of expression of the protein in normal murine embryonic tissues. The analysis of different organs of *Slc25a1* wild-type embryos by immunoblot showed strong expression in the brain, heart, lung, liver, colon spleen and pancreas (Fig. 1B). This finding was confirmed with immunohistochemistry (Fig. 1C, D). Immunofluorescence of the head showed specific enrichment of SLC25A1 protein in the mitochondria, as revealed by co-staining with mitofilin (Supplementary Fig. 1A, B). In addition, within the craniofacial region, SLC25A1 signal was particularly strong in the frontal region, in the mouth, eyes, palate and tongue, as well as in the brain and cerebellum, all sites affected in human diseases sustained by its deficiency (Fig. 1C and Supplementary Fig. 1A).

**Fig. 2:**
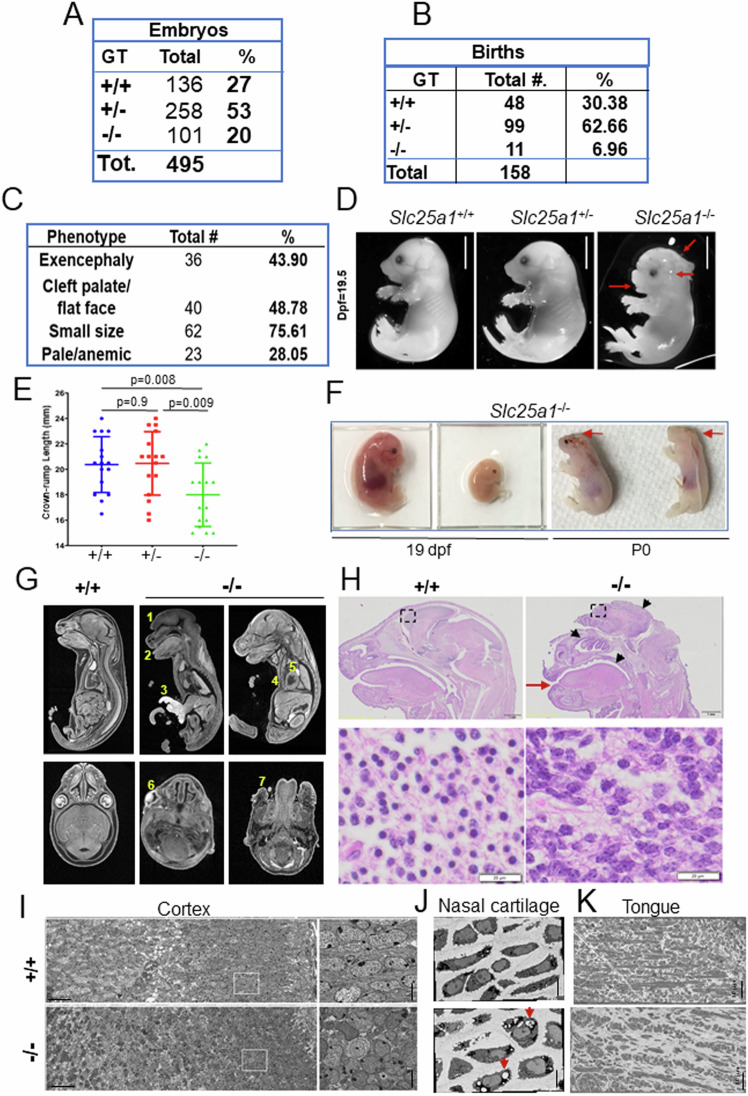
The knock-out of the *Slc25a1* gene leads to perinatal lethality. **A** Representation of the genotypes observed across 85 litters (495 mice) obtained by breeding *Slc25a1* heterozygous mice. **B** Number and percentage of births observed across a population of newborn +/+, +/- and -/- mice. **C** Prevalence of the phenotypic alterations observed in *Slc25a1*^-/-^ embryos. **D** Representative images of *Slc25a1*^+/+^, *Slc25a1*^+/-^ and *Slc25a1*^-/-^ embryos under stereoscopic microscope at E18.5 dpf. Red arrows point to cleft palate, underdeveloped ears, and brain abnormalities. Bar = 5 mm. **E** Measurements of crows to rump length showing smaller body size in *Slc25a1* homozygous mice compared with wild-type and heterozygous littermates (*n* = 16 mice per genotype, respectively). *p*-values were calculated with two-tailed non-parametric t-test. **F** Heterogeneity of the phenotypes of *Slc25a1*^-/-^ embryos at 19pdf or at P0, showing variations in body size, lack of skull and eyes in the last two born embryos on left (indicated by arrows). **G** Computed Tomography (CT) scan of wild-type or *Slc25a1*^-/-^ embryos at E18.5 dpf. Yellow numbers indicate: 1: Exencephaly; 2: Protruding tongue; 3: Gastroschisis; 4: Pectus excavatum; 5: Abnormal heart. 6: Abnormal eye; 7: Abnormal facial area morphology. Bar = 1 mm. **H** Representative images of H&E staining in the embryos of the indicated genotype. Top panels show the anatomy of the whole head; lower panels show magnification of the cortical region and nuclear abnormalities in *Slc25a1*^-/-^ mice. Bar = 20 µm. Red arrow points to hypertrophic tongue. Scanning electron microscopy (SEM) overviews of the neocortex (**I**), nasal cartilage (**J**) and tongue (**K**) in wild-type and *Slc25a1*^-/-^ embryos at E18.5 dpf. Arrows in J point to vacuolization in the nasal cartilage.

To understand the impact of SLC25A1 activity on embryonic development, we generated a knock-out mouse model. Mice harboring a heterozygous germline targeted deletion in one *Slc25a1* allele were described by our group before [27]. The targeting vector is inserted between intron *1* and *2* of the *Slc25a1* gene on chromosome 16. We first expanded a population of heterozygous *Slc25a1*^*+/-*^ mice, and intercrosses of these animals subsequently allowed studying the population of homozygous mutants. These null mice did not react with the anti-SLC25A1 antibody in immunofluorescence (Supplementary Fig. 1B) and lacked the SLC25A1 protein and mRNA in all tissues examined (Fig. 1E, and Supplementary Fig. 2A–C). Relative to their wild-type and heterozygous counterparts, *Slc25a1* null mice were detected at roughly normal Mendelian ratio (Fig. 2A). Few of them were also born, succumbing shortly after birth (Fig. 2B). Importantly, these nullizygous embryos were detected at all stages of development from day 7 to day 19-21 dpf (Supplementary Fig. 2D), indicating that the knock-out of the *Slc25a1* gene leads to a perinatal lethal phenotype, but is not overtly embryonic lethal. A substantial spectrum of alterations affecting multiple organs was detected in these mice. The majority of them displayed small body size and anemia/pallor (Fig. 2C, E), malformation/absence of the ears, absence of one or both eyes, as well as cleft palate (Fig. 2D, F). The cranio-facial region and the brain were the most affected and 43% of embryos showed overt defects in neural tube closure, manifested as exencephaly or anencephaly. A repository of computed tomography (CT) images available through the mouse phenotype consortium at UC Davis [28, 29], confirmed that the craniofacial region of *Slc25a1*^*-/-*^ embryos was severely dysmorphic, including bulging or absent eyes, dilated turbinates, enlarged and protruding tongue (Fig. 2G and Supplementary Fig. 3A). Brain and liver hemorrhage, heart alterations, gastroschisis (Fig. 2G^3^ and Supplementary Fig. 3B), and bone abnormalities were also present. These latter were significant and include lack of skull (Fig. 2G, F), pectus excavatum (Fig. 2G^4^), abnormal curvatures and thickness of the spine and increased cartilage content in the vertebral column reminiscent of enchondromatosis (Fig. 2G^3^ and Supplementary Fig. 3C, D). H&E staining and scanning electron microscopy (SEM) performed on the brain and craniofacial region further revealed prominent disorganization in the developing neocortex of *Slc25a1*^*-/-*^ embryos at E18 dpf, showing diffused areas of nuclear pleomorphism with alterations in nuclear size, disorganization of the stroma, and mis-orientation of neurons in the distinctive cortical layers (Fig. 2H, I). Alterations in the mitochondrial morphology were also seen (not shown). The comparison of chondroblasts and collagen from the nose cartilage revealed morphological differences, with an increase in cytoplasmic vacuolization seen in *Slc25a1* deficient animals, and disruption in the directionality of collagen fibers in the matrix (Fig. 2J). In addition, the tongue of virtually all *Slc25a1*^*-/-*^ mice appeared abnormal in size and protruding, with thicker myocytes, altered directionality of fiber packages and increased connective tissue (Fig. 2H, K). By contrast, *Slc25a1* heterozygous showed no obvious defects and developed normally.

Thus, systemic loss of the *Slc25a1* alleles during embryonic development in the mouse recapitulates but also severely expands the spectrum of alterations seen in human DGS/VCFS and in D/L-2HGA.

### SLC25A1 deficient embryos undergo metabolic and transcriptional rewiring

**Fig. 3:**
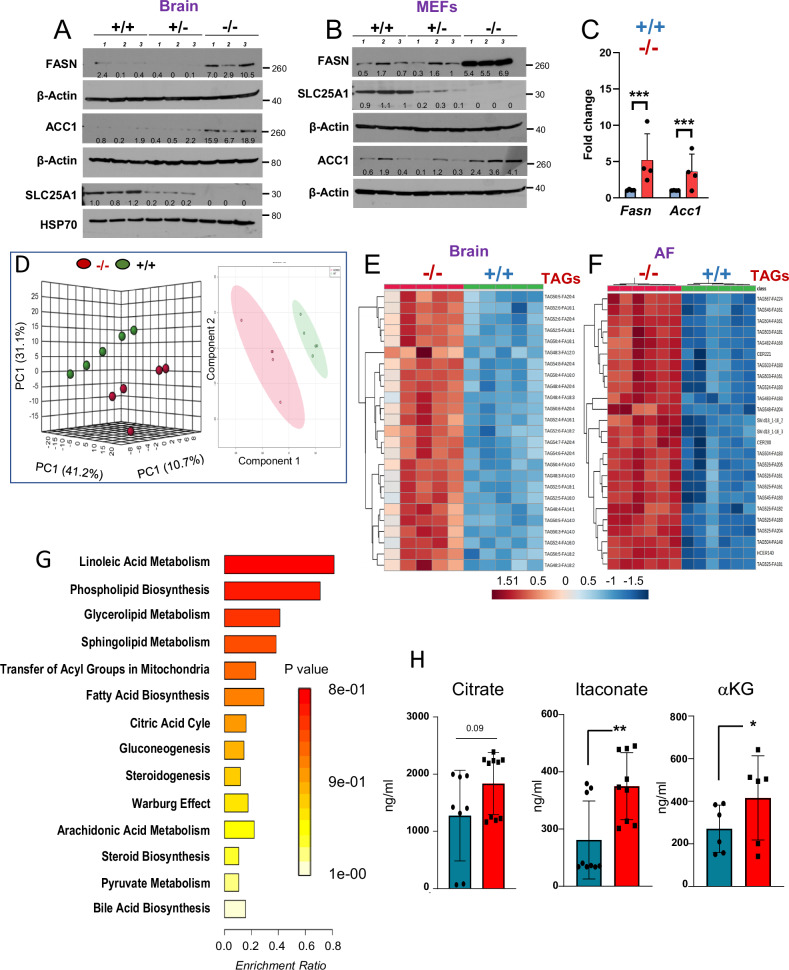
Loss of SLC25A1 induces metabolic remodeling. **A-B** Expression of the indicated proteins in brain (**A**) and MEFs (**B**) isolated from *Slc25a1*^+/+^, *Slc25a1*^+/-^ and *Slc25a1*^-/-^ embryos (*n* = 3 per genotype). **C** mRNA levels detected with q-RT-PCR of the indicated genes in MEFs *Slc25a1*^+/+^ (blue) and *Slc25a1*^-/-^ (red). Each dot represents MEFs isolated from a different mouse (*n* = 4). **D** Principal component analysis (PCA) scores plot and the partial least squares (PLS) of 5 wild-type and *Slc25a1* nullyzygous embryos used for metabolic studies. **E-F** Heatmap of top 25 enriched lipids in brain and AF samples obtained from *Slc25a1*^+/+^ (green) and *Slc25a1*^-/-^ (red) (*n* = 5, from different litters, each in triplicate). Normalization was performed by dividing the area under the curve for each metabolite by the internal standard area. Clustering was performed by using the Metaboanalyst software. **G** Pathway enrichment analysis in the amniotic fluids obtained from *Slc25a1*^-/-^ embryos relative to wild-type (*n* = 3 per genotype, each in 3 technical replicates). **H** Concentrations of the indicated TCA intermediates in the amniotic fluid obtained from *Slc25a1*^+/+^ and *Slc25a1*^-/-^ embryos (*n* = 3-5, each in technical replicates). All data are presented as mean value ± SD. Unpaired non-parametric t-test was used throughout. In all blots, quantification was performed using ImageJ normalized to housekeeping protein (β-Actin or Hsp70), and results are presented as a fold change relative to control wild type.

The current model envisions that the pathogenesis of diseases due to impaired SLC25A1 activity is sustained by the deficit of citrate resulting in impairment of lipids. We tested this model by performing targeted and untargeted metabolomic and lipidomic analyses, paralleled by immuno-blot experiments interrogating the main pathways involved in lipid biosynthesis. Surprisingly, the levels of key rate-limiting lipogenic enzymes, fatty acid synthase (FASN) and acetyl-CoA carboxylase Alpha (ACC1) were up-regulated in the brains and MEFs derived from *Slc25a1*^*-/-*^ mice, at both the protein and mRNA levels (Fig. 3A–C). Global metabolomic analysis showed a good separation between these samples, with the homozygous null mice clustering together (Fig. 3D). Surprisingly, the main alteration detected in the brains and amniotic fluids of *Slc25a1* null mice, consisted of enhanced triglyceride species (Fig. 3E, F), enrichment in linoleic acid, phospholipids, glycerolipids, sphingolipids, arachidonic-, steroid-, and bile acid biosynthetic pathway (Fig. 3G). The concentration of αKG, the precursor of 2HG, and of citrate were elevated, and itaconate, which is derived from cytosolic citrate *via* the activity of Immune-Responsive Gene 1, *IRG1*, was significantly increased (Fig. 3H).

**Fig. 4:**
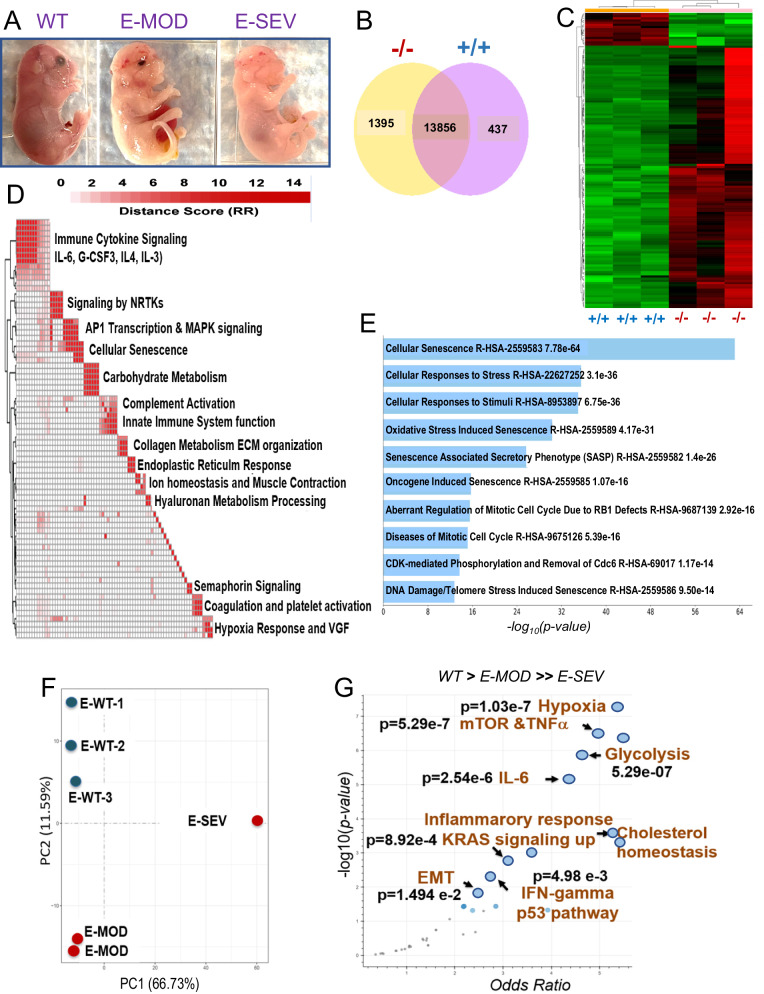
Transcriptional analysis of *Slc25a1* deficient embryos. **A** Representative phenotypes of *Slc25a1*^*-/-* .^mice classified based on the severity of the phenotype (E18-19 dpf). **B** Venn diagram representing genes differentially regulated in the brains isolated from *Slc25a1*^+/+^ and *Slc25a1*^-/-^ embryos at 18.5 dpc (*n* = 3). **C** Hierarchical clustering map of differentially expressed genes as in B. **D** Pathway enrichment analysis of genes significantly elevated in *Slc25a1*^-/-^ mice relative to wild-type. Enriched pathways (FDR < 0.05), assessed from the Reactome Database, were clustered *via* a distance metric derived from shared significant genes common amongst enriched gene sets. Clustered gene sets were summarized by biological function. **E** Main pathways related to senescence induced in *Slc25a1*^*-/-*^ embryos detected with Enrichr (Reactome 2022). *p*-values are generated by the software and are indicated. **F** PCA plot of the transcriptomic profiles of indicated embryos. **G** Transcriptomic data derived from each of the embryos were used for a Hue-Saturation-Value (HSV) analysis. The Enrichr gene set tool was employed to identify gene sets that are significantly more active in E-SEV and moderately elevated in E-MOD *versus* wild-type (*WT*. > *E-MD*. > > *E-SV);* *p* < 0.05.

We next conducted transcriptome analysis using *Slc25a1*^*-/-*^ mice with varying phenotypic alterations, classified as severe (E-SEV) or moderate (E-MOD) (Fig. 4A, F). E-SEV mice displayed critical abnormalities, including severe facial deformities, anophthalmia, cleft palate, transparent skin and severe brain alterations. Global transcriptome and hierarchical clustering identified 1395 genes uniquely regulated in *Slc25a1* homozygous mutants (Fig. 4B, C), which included: genes involved in inflammatory and pro-oncogenic pathways (Immune Cytokine Signaling, Signaling by NRTKs, AP1 Transcription & MAPK Signaling), the hypoxia response and, noticeably, induction of senescence and of several senescence-associated pathways (Fig. 4D, E, Supplementary Fig. 4A and Table 1). Using the Enrichr gene set tool [30, 31], we determined that the top up-regulated signals in the E-SEV were the hypoxia response, along with pro-inflammatory (TNFα, IL-6) and pro-oncogenic pathways, including mTORC, the KRas pathways, and the induction of the Epithelial to Mesenchymal Transition (EMT) (Fig. 4G). Additionally, both glycolysis and cholesterol responses were enhanced, in agreement with the previous metabolic analysis. Interestingly, *Slc25a1*^*-/-*^ embryos also displayed an enrichment of tumor-associated transcriptional signatures involved in breast cancer, neuroblastoma, ovarian cancer, and others, as well as induction of the p53 tumor suppressor (Supplementary Fig. 4B).

**Table 1:** a: Genes related to senescence and cell cycle regulated in Slc25a1*-/-* brains. Significantly up-regulated or down-regulated genes (FDR < 0.05) related to senescence were assessed for pathway enrichment using the Reactome 2022 via the EnrichR software. b. Significantly up- or down-regulated genes as in 1a, were assessed with Go Biological Processes using the Enrichr software.

a			
Upregulated in KO versus WT (Reactome 2022)	*p*-value	*q*-value	Overlap genes
NAME			
Cellular Senescence R-HSA-2559583	7.78E-64	4.50E-61	[CEBPB, CDKN1A, ANAPC15, ETS1, ETS2, ANAPC11, UBN1, RPS6KA1, RBBP7, RPS27A, MAPK3, CBX8, KDM6B, SUZ12, MAP2K3, CDKN2D, JUN, CDKN2C, RING1, CBX4, ANAPC7, UBE2C, TINF2, HMGA1, UBE2E1, HMGA2, FOS, MAPK14, TERF1, CCNA2, CDK6, MAPKAPK3, CCNE2, CCNE1, ID1, MAPKAPK2, CDK2, MAPKAPK5, MDM4, UBA52, EZH2]
Cellular Responses To Stress R-HSA-2262752	3.10E-36	8.97E-34	[CEBPB, CDKN1A, ANAPC15, ETS1, ETS2, ANAPC11, UBN1, RPS6KA1, RBBP7, RPS27A, MAPK3, CBX8, KDM6B, SUZ12, MAP2K3, CDKN2D, JUN, CDKN2C, RING1, CBX4, ANAPC7, UBE2C, TINF2, HMGA1, UBE2E1, HMGA2, FOS, MAPK14, TERF1, CCNA2, CDK6, MAPKAPK3, CCNE2, CCNE1, ID1, MAPKAPK2, CDK2, MAPKAPK5, MDM4, UBA52, EZH2]
Cellular Responses To Stimuli R-HSA-8953897	6.75E-36	1.30E-33	[CEBPB, CDKN1A, ANAPC15, ETS1, ETS2, ANAPC11, UBN1, RPS6KA1, RBBP7, RPS27A, MAPK3, CBX8, KDM6B, SUZ12, MAP2K3, CDKN2D, JUN, CDKN2C, RING1, CBX4, ANAPC7, UBE2C, TINF2, HMGA1, UBE2E1, HMGA2, FOS, MAPK14, TERF1, CCNA2, CDK6, MAPKAPK3, CCNE2, CCNE1, ID1, MAPKAPK2, CDK2, MAPKAPK5, MDM4, UBA52, EZH2]
Oxidative Stress Induced Senescence R-HSA-2559580	4.17E-31	6.04E-29	[CBX8, KDM6B, SUZ12, MAP2K3, CDKN2D, JUN, CDKN2C, RING1, CBX4, FOS, MAPK14, CDK6, MAPKAPK3, MAPKAPK2, MAPKAPK5, MDM4, RBBP7, RPS27A, UBA52, EZH2, MAPK3]
Senescence-Associated Secretory Phenotype (SASP) R-HSA-2559582	1.40E-26	1.62E-24	[CDKN2D, CDKN1A, CEBPB, JUN, ANAPC15, CDKN2C, ANAPC7, UBE2C, UBE2E1, FOS, ANAPC11, CCNA2, CDK6, RPS6KA1, CDK2, RPS27A, UBA52, MAPK3]
Oncogene Induced Senescence R-HSA-2559585	1.07E-16	1.04E-14	[CDKN2D, CDKN2C, CDK6, ID1, MDM4, RPS27A, ETS1, UBA52, ETS2, MAPK3]
Aberrant Regulation Of Mitotic Cell Cycle Due To RB1 Defects R-HSA-9687139	2.92E-16	2.42E-14	[CDKN1A, ANAPC15, CDK6, ANAPC7, CCNE2, UBE2C, CCNE1, CDK2, UBE2E1, ANAPC11]
Diseases Of Mitotic Cell Cycle R-HSA-9675126	5.39E-16	3.90E-14	[CDKN1A, ANAPC15, CDK6, ANAPC7, CCNE2, UBE2C, CCNE1, CDK2, UBE2E1, ANAPC11]
CDK-mediated Phosphorylation And Removal Of Cdc6 R-HSA-69017	1.17E-14	7.53E-13	[CCNA2, ANAPC15, ANAPC7, CCNE2, CCNE1, UBE2C, CDK2, UBE2E1, RPS27A, UBA52, ANAPC11]
DNA Damage/Telomere Stress Induced Senescence R-HSA-2559586	9.50E-14	5.50E-12	[CCNA2, CDKN1A, CCNE2, CCNE1, TINF2, UBN1, CDK2, HMGA1, HMGA2, TERF1]
**Downregulated in KO versus WT (Reactome 2022)**			
Cellular Senescence R-HSA-2559583	8.23E-105	4.73E-102	[CDKN1B, EHMT2, UBE2D1, EHMT1, CABIN1, LMNB1, RPS6KA3, CDC23, KAT5, CDC26, RPS6KA2, CDC27, SCMH1, MAP3K5, MAP2K4, EED, MINK1, TERF2, CCNA1, TERF2IP, ANAPC4, ANAPC5, TNIK, ASF1A, ANAPC1, ANAPC2, ANAPC16, RNF2, ANAPC10, HIRA, MAPK9, FZR1, MAPK8, MAPK7, RBBP4, UBB, EP400, MAPK1, MAP2K7, MAP2K6, MAP4K4, ACD, CBX6, PHC2, CDKN2B, PHC1, CBX2, PHC3, MAPK10, MAPK11, CDK4, CDC16, MDM2, ATM]
Cellular Responses To Stress R-HSA-2262752	5.84E-67	1.68E-64	[CDKN1B, EHMT2, UBE2D1, EHMT1, CABIN1, LMNB1, RPS6KA3, CDC23, KAT5, CDC26, RPS6KA2, CDC27, SCMH1, MAP3K5, MAP2K4, EED, MINK1, TERF2, CCNA1, TERF2IP, ANAPC4, ANAPC5, TNIK, ASF1A, ANAPC1, ANAPC2, ANAPC16, RNF2, ANAPC10, HIRA, MAPK9, FZR1, MAPK8, MAPK7, RBBP4, UBB, EP400, MAPK1, MAP2K7, MAP2K6, MAP4K4, ACD, CBX6, PHC2, CDKN2B, PHC1, CBX2, PHC3, MAPK10, MAPK11, CDK4, CDC16, MDM2, ATM]
Cellular Responses To Stimuli R-HSA-8953897	1.70E-66	3.26E-64	[CDKN1B, EHMT2, UBE2D1, EHMT1, CABIN1, LMNB1, RPS6KA3, CDC23, KAT5, CDC26, RPS6KA2, CDC27, SCMH1, MAP3K5, MAP2K4, EED, MINK1, TERF2, CCNA1, TERF2IP, ANAPC4, ANAPC5, TNIK, ASF1A, ANAPC1, ANAPC2, ANAPC16, RNF2, ANAPC10, HIRA, MAPK9, FZR1, MAPK8, MAPK7, RBBP4, UBB, EP400, MAPK1, MAP2K7, MAP2K6, MAP4K4, ACD, CBX6, PHC2, CDKN2B, PHC1, CBX2, PHC3, MAPK10, MAPK11, CDK4, CDC16, MDM2, ATM]
Oxidative Stress Induced Senescence R-HSA-2559580	1.29E-42	1.85E-40	[RNF2, MAPK9, MAPK8, RBBP4, UBB, SCMH1, MAPK1, MAP2K7, MAP3K5, MAP2K6, MAP4K4, MAP2K4, CBX6, PHC2, CDKN2B, PHC1, EED, CBX2, MINK1, PHC3, MAPK10, MAPK11, CDK4, MDM2, TNIK]
Senescence-Associated Secretory Phenotype (SASP) R-HSA-2559582	9.17E-40	1.05E-37	[CDKN2B, CDKN1B, ANAPC16, EHMT2, UBE2D1, EHMT1, ANAPC10, CCNA1, RPS6KA3, FZR1, CDC23, MAPK7, UBB, CDK4, CDC26, RPS6KA2, CDC16, CDC27, ANAPC4, MAPK1, ANAPC5, ANAPC1, ANAPC2]
Aberrant Regulation Of Mitotic Cell Cycle Due To RB1 Defects R-HSA-9687139	1.49E-26	1.42E-24	[CDKN1B, ANAPC16, UBE2D1, ANAPC10, FZR1, CDC23, CDK4, CDC26, CDC27, CDC16, ANAPC4, ANAPC5, ANAPC1, ANAPC2]
Diseases Of Mitotic Cell Cycle R-HSA-9675126	3.77E-26	3.10E-24	[CDKN1B, ANAPC16, UBE2D1, ANAPC10, FZR1, CDC23, CDK4, CDC26, CDC27, CDC16, ANAPC4, ANAPC5, ANAPC1, ANAPC2]
Transcriptional Regulation By VENTX R-HSA-8853884	5.86E-26	4.09E-24	[ANAPC16, EHMT2, EHMT1, UBE2D1, ANAPC10, FZR1, CDC23, CDC26, CDC27, CDC16, ANAPC4, ANAPC5, ANAPC1, ANAPC2]
Conversion From APC/C:Cdc20 To APC/C:Cdh1 In Late Anaphase R-HSA-176407	7.12E-26	4.09E-24	[FZR1, CDC23, ANAPC16, CDC26, CDC16, CDC27, UBE2D1, ANAPC4, ANAPC5, ANAPC10, ANAPC1, ANAPC2]
Aberrant Regulation Of Mitotic Exit In Cancer Due To RB1 Defects R-HSA-9687136	7.12E-26	4.09E-24	[FZR1, CDC23, ANAPC16, CDC26, CDC16, CDC27, UBE2D1, ANAPC4, ANAPC5, ANAPC10, ANAPC1, ANAPC2]

b			
Upregulated in KO versus WT (Go Biological Processes 2023)			
NAME	*p*-value	q-value	Overlap genes
Regulation Of Protein Serine/Threonine Kinase Activity (GO:0071900)	1.56E-08	8.00E-06	[CDKN2D, CCNA2, CDKN1A, CDKN2C, CCNE2, CCNE1, HMGA2, EZH2]
Cell Cycle G1/S Phase Transition (GO:0044843)	5.95E-08	1.20E-05	[CCNA2, CDKN1A, CDK6, CCNE2, CCNE1, CDK2]
Regulation Of Cyclin-Dependent Protein Kinase Activity (GO:1904029)	6.69E-08	1.20E-05	[CCNA2, CDKN2D, CDKN1A, CDKN2C, CCNE2, CCNE1]
Mitotic Cell Cycle Phase Transition (GO:0044772)	3.05E-07	4.10E-05	[CCNA2, CDKN1A, CDK6, CCNE2, UBE2C, CCNE1, CDK2]
Regulation Of Cyclin-Dependent Protein Serine/Threonine Kinase Activity (GO:0000079)	7.46E-07	8.10E-05	[CCNA2, CDKN2D, CDKN1A, CDKN2C, CCNE2, CCNE1]
Regulation Of Mitotic Cell Cycle Phase Transition (GO:1901990)	1.23E-06	1.04E-04	[CDKN2D, CDKN1A, CDK6, CDKN2C, UBE2C, CDK2]
Anaphase-Promoting Complex-Dependent Catabolic Process (GO:0031145)	1.35E-06	1.04E-04	[ANAPC15, ANAPC7, UBE2C, ANAPC11]
Stress-Induced Premature Senescence (GO:0090400)	2.13E-06	1.44E-04	[CDKN1A, MAPKAPK5, MAPK14]
G1/S Transition Of Mitotic Cell Cycle (GO:0000082)	5.29E-06	3.17E-04	[CDKN1A, CDK6, CCNE2, CCNE1, CDK2]
Regulation Of Mitotic Metaphase/Anaphase Transition (GO:0030071)	4.39E-05	2.26E-03	[ANAPC15, ANAPC7, UBE2C, ANAPC11]
**Downregulated in KO versus WT (Go Biological Processes 2023)**			
NAME	***p*-value**	***q*-value**	Overlap genes
Regulation Of Cell Cycle (GO:0051726)	3.36E-21	2.08E-18	[CDKN1B, ANAPC16, RNF2, ANAPC10, FZR1, CDC23, KAT5, CDK4, CDC26, RPS6KA2, CDC16, CDC27, EP400, MDM2, ANAPC4, ATM, ANAPC5, ANAPC1, ANAPC2, MAP2K6]
Regulation Of Meiotic Cell Cycle (GO:0051445)	1.35E-20	4.17E-18	[FZR1, CDC23, ANAPC16, CDC26, CDC16, CDC27, ANAPC4, ANAPC5, ANAPC10, ANAPC2]
Anaphase-Promoting Complex-Dependent Catabolic Process (GO:0031145)	2.57E-20	5.29E-18	[FZR1, CDC23, ANAPC16, CDC26, CDC16, CDC27, ANAPC4, ANAPC5, ANAPC10, ANAPC2]
Stress-Activated MAPK Cascade (GO:0051403)	1.02E-16	1.57E-14	[MAPK10, MAPK9, MAP2K4, MAPK11, MAPK8, MINK1, MAPK1, MAP2K7, MAP2K6, MAP3K5]
Regulation Of Mitotic Cell Cycle (GO:0007346)	1.81E-16	2.24E-14	[CDKN1B, ANAPC16, ANAPC10, FZR1, CDC23, CDC26, CDC16, CDC27, MDM2, ANAPC4, ANAPC5, ANAPC1, ANAPC2]
Protein K11-linked Ubiquitination (GO:0070979)	2.50E-16	2.57E-14	[FZR1, CDC23, CDC26, CDC27, CDC16, ANAPC4, ANAPC5, ANAPC10, ANAPC2]
MAPK Cascade (GO:0000165)	8.38E-13	7.40E-11	[MAPK11, MAP2K4, MAPK8, MINK1, MAPK1, TNIK, MAP2K7, MAP3K5, MAP2K6, MAP4K4]
JNK Cascade (GO:0007254)	4.52E-12	3.50E-10	[MAPK10, MAPK9, MAP2K4, MAPK8, MINK1, MAP2K7, MAP3K5]
Protein Polyubiquitination (GO:0000209)	9.30E-12	6.39E-10	[FZR1, CDC23, CDC26, CDC16, CDC27, MDM2, UBE2D1, ANAPC4, ANAPC5, ANAPC1, ANAPC10, ANAPC2]
Proteasome-Mediated Ubiquitin-Dependent Protein Catabolic Process (GO:0043161)	3.24E-11	2.01E-09	[ANAPC16, ANAPC10, FZR1, CDC23, KAT5, CDC26, CDC16, CDC27, MDM2, ANAPC4, ANAPC5, ANAPC1, ANAPC2]

We conclude that in this mouse model, SLC25A1 is not rate limiting for the maintenance of the citrate and lipid pool as generally thought, also consistent with the existence of several pathways able to replenish cytosolic citrate in cells lacking SLC25A1, including *via* glutamine reductive carboxylation [3, 32, 33]. Instead, SLC25A1 loss rewires the transcriptome towards senescence, pro-oncogenic and pro-inflammatory signals and induces a hypoxic response.

### SLC25A1 loss induces MiDAS and an OIS-like patterns of senescence

**Fig. 5:**
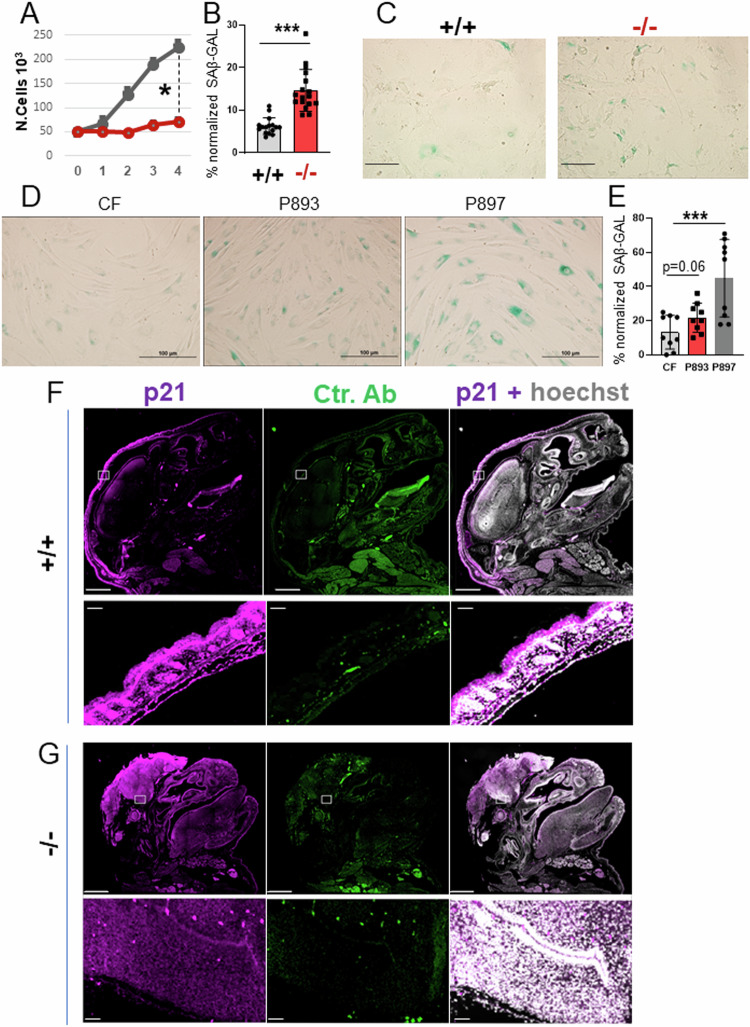
SLC25A1 dysfunction induces premature senescence. **A** Growth curves of MEF cells isolated from *Slc25a1*^+/+^ and *Slc25a1*^-/-^ embryos. Cells were plated at the same density (5000 cells per well) and counted over the course of several days using trypan blue exclusion. Quantification of the SA-β*-*GAL positive MEF cells (%) normalized to the total number of cells identified with DAPI counter-staining pooled from two independent experiments (mean value ± SD) (**B**) and representative images of (SA-β*-*GAL) activity (**C**). Representative images of SA-β*-*GAL staining in human fibroblasts (**D**) and quantification of the SA-β*-*GAL positive cells normalized to the total number of cells identified with DAPI counter-staining (**E**). All IF data are presented as % positive cells ± SD pooled from two independent experiments. Bar = 100 μm. p21 immunofluorescence staining of the head of *Slc25a1*^+/+^ (**F**) and *Slc25a1*^-/-^ (**G**) embryos at E19 dpf. Hoechst was used to counterstain the nuclei. Secondary antibody alone was used as a control. Bar = 1000 µM. Squares indicate areas of interests enlarged in each of the lower panels.

Next, we examined the growth properties of embryo fibroblasts (MEFs) derived from mice, as well as skin fibroblasts obtained from two young patients affected by D/L-2HG-aciduria, patient 893 and patient 897 [34], and we found that they both exhibited a proliferation defect at early passages (Fig. 5A and not shown). This loss of proliferative capacity was suggestive of premature senescence, which when aberrantly activated during embryonic development drives neural tube closure defects (NTDs) and is strongly associated with maternal diabetes and with dysregulation of the folate metabolic pathway that controls DNA replication, indicating a key role for metabolic alterations in the abnormal development of the neural tube [35–37]. At a cellular level, senescence is identified by enhanced beta-galactosidase *(*SA-β*-*GAL) activity at low pH, by breakdown and loss of nuclear lamin B1, as well as by chromatin-dense structures in the nucleus called the Senescence-associated heterochromatin foci (SAHF) [35]. The *Slc25a1*^*-/-*^ MEFs as well as the patients fibroblasts showed a strong SA-β*-*GAL signal (Fig. 5B-E), a significant increase in the number of the SAHF (Supplementary Fig. 5A, B), as well as depletion of lamin B1 by both immuno-blot and qRT-PCR (Supplementary Fig. 5C and not shown). In addition, the head and brain of *Slc25a1*^*-/-*^ mice harvested at late development stage (E19 dpf), reacted with antibodies directed against p21, one of the key mediators of senescence (Fig. 5F, G). To ascertain that induction of senescence is a direct consequence of impairment of SLC25A1 activity, we employed a specific shRNA as well as the specific inhibitor (SLC25A1-I, Ref. [3]). With both these approaches we detected a strong induction of SAβ*-*GAL activity and arrest of the cell cycle (Supplementary Fig. 5D, E), demonstrating a direct effect of SLC25A1 inhibition in the induction of senescence.

**Fig. 6:**
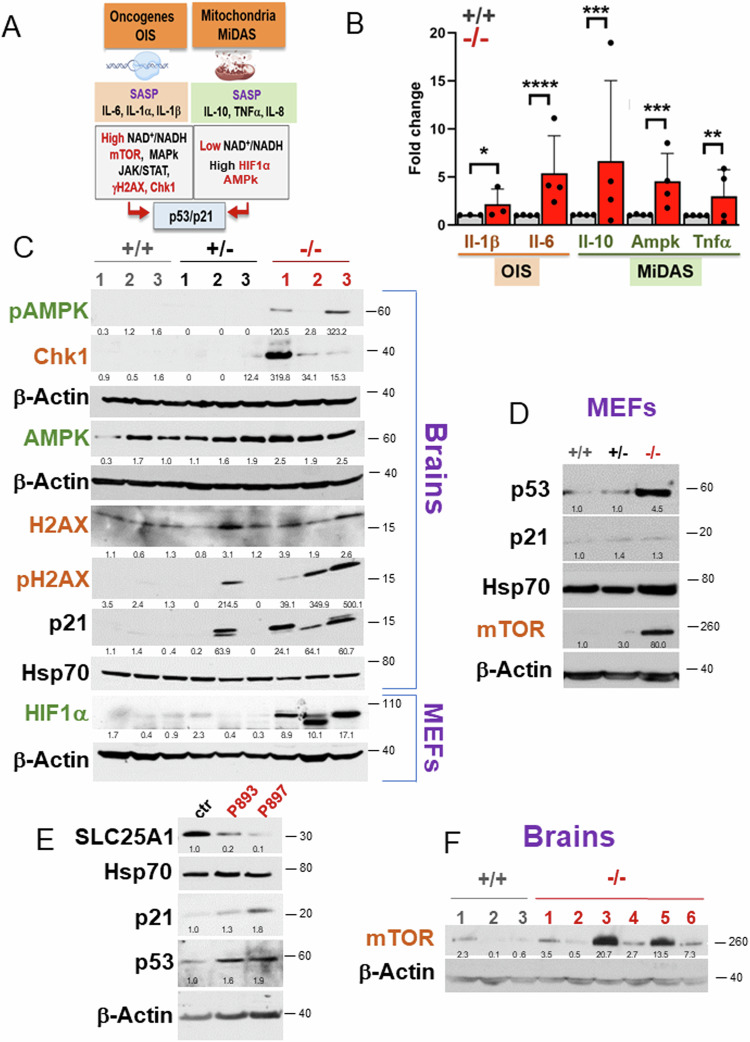
The OIS-*lp* and MiDAS pathways of senescence are induced when SLC25A1 is lost. **A** Scheme of the OIS and MiDAS pathways of senescence and of the associated SASPs, depicting DNA replication stress or mitochondrial dysfunction, respectively (see also text for explanation) (*created with Biorender*). **B** mRNA levels of the OIS (brown) and MiDAS (green) SASPs in *Slc25a1*^+/+^ (grey) and *Slc25a1*^*-*/-^ (red) MEFs, respectively (*n* = 3–4). Expression level of the indicated proteins involved in OIS (brown) and MiDAS (green) in the brain samples (**C**, **F**), MEFs (**C**, **D**) and in the patient’s fibroblasts (**E**) (*n* = 1–6). In all blots, quantification was performed using ImageJ normalized to housekeeping protein (β-Actin or Hsp70), and results are presented as a fold change relative to control (either +/+ mice or control fibroblasts).

Given the existence of different pathways and types of SASP associated with senescence (summarized in Fig. 6A), we next sought to identify the specific signals elicited by SLC25A1 loss. In both the *Slc25a1*^*-/-*^ MEFs and in the patient-derived fibroblasts, we detected a dramatic increase in markers associated with MiDAS, such as IL-10, TNFα and AMPk, as well as of IL-1β, IL-6, γH2AX, Chk1 and mTOR, which are indicative of OIS (Fig. 6B–D). We refer to this latter pathway of senescence, as OIS-like pattern of senescence (or OIS-*lp*), to indicate the absence of an obvious canonical oncogene product as the underlying mechanism. Further, the levels of HIF1α were increased in cells cultured in vitro in normoxic conditions, suggestive of a pseudo-hypoxia response (Fig. 6C), and p53 and p21 were induced in both the *Slc25a1* deficient mice and in the patient-derived fibroblasts (Fig. 6C–E). Consistent with the previous transcriptomic data, the expression of mTORC, which plays a role not only in nutrient sensing but also as a mediator of senescence [38], were elevated in most of these brains (Fig. 6F).

Thus, loss of SLC25A1 leads to the simultaneous activation of both an OIS-*lp* and MiDAS pathway, prompting us to investigate the molecular mechanisms by which these responses are induced.

### SLC25A1 dysfunction impairs OXPHOS *via* depletion of NAD^+^ and enhanced PDK1-mediated glycolysis

**Fig. 7:**
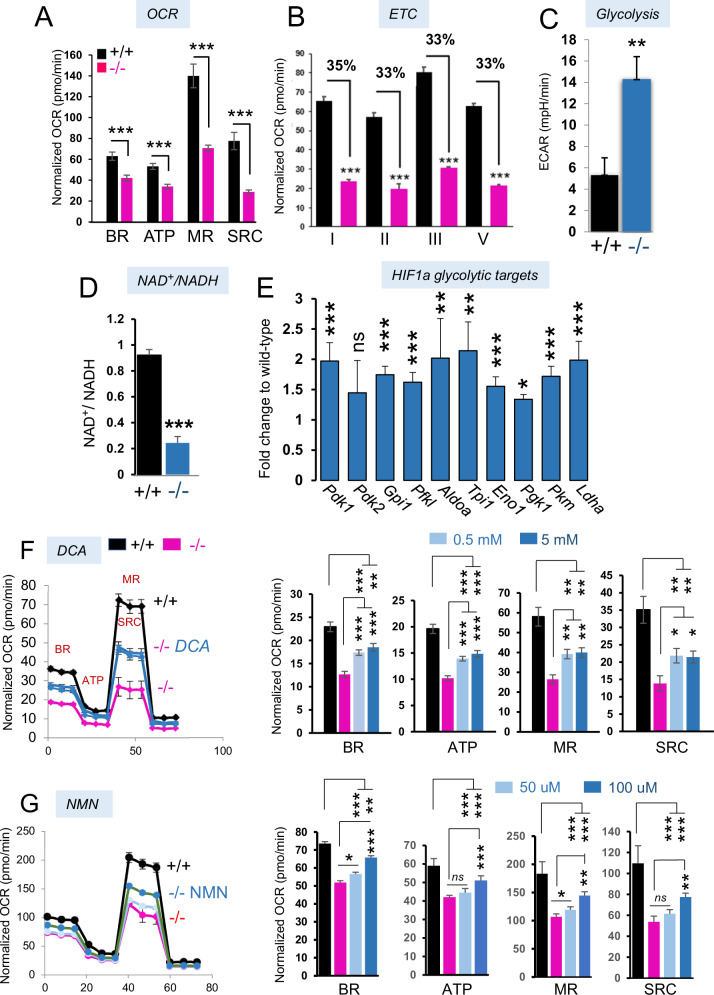
NAD^+^ and ETC impairment in cells with dysfunctional SLC25A1. **A** Oxygen consumption rate (OCR) was assessed using Seahorse Extracellular Flux Analyzer in MEFs isolated from *Slc25a1*^+/+^ (black) and *Slc25a1*^*-*/-^ (pink) embryos. Injection of Oligomycin, FCCP and Rotenone/Antimycin allowed calculation of the BR= Basal respiration; ATP= mitochondrial ATP Production, MR= Maximal Respiration, SRC= Spare Respiratory Capacity. **B** Activities of the indicated ETC complexes measured in +/+ or -/- MEFs. % = residual activity. **C** Glycolysis in the indicated MEFs measured as Extracellular acidification (ECAR). **D** NAD^+^/NADH ratio in MEFs isolated from *Slc25a1*^+/+^ and *Slc25a1*^*-*/-^ embryos. **E** Relative expression of the indicated HIF1α genes/transcripts in brains isolated from *Slc25a1*^+/+^ and *Slc25a1*^*-*/-^ embryos (*n* = 3). OCR measured in +/+ or -/- MEFs untreated or treated with the indicated concentration of DCA (**F**) or NMN (**G**). Bars represent SEM and *p*-values were calculated using two tailed non parametric t-test. Experiments shown in this Figure were performed multiple times (2-3).

The NAD^+^/NADH ratio is a key determinant of OXPHOS. Several steps in the TCA cycle generate NADH from NAD+ and this chain of reactions is necessary for respiration *via* the activity of Complex-I (CI), which donates the electrons derived from NADH to other respiratory complex subunits within the electron transport chain (ETC). First, we determined that the *Slc25a1*^*-/-*^ MEFs exhibited a severe deficit in the basal (BR), maximal (MR) and spare respiratory capacity (SRC) and ATP production (Fig. 7A), coinciding with a significant loss of function of complex I, II and III and V (Fig. 7B). A similar respiratory deficit was recently described in the patient fibroblasts [34]. In addition, as we observed previously in tumor cells [18], MEFs cells lacking SLC25A1 also prominently turned on glycolysis (Fig. 7C), which consumes NAD^+^ because many glycolytic enzymes use NAD^+^ as a co-factor [39]. Accordingly, the NAD^+^/NADH ratio was significantly reduced in the *Slc25a1*^*-/-*^ MEFs (Fig. 7D).

We then focused on the pseudo-hypoxia response, which is known to drive glycolysis. Consistent with prior data, the brains of *Slc25a1*^*-/-*^ mice were enriched in glycolytic genes that are under direct transcriptional control of HIF1α. These included the kinase PDK1, glucose transporters (MCT4, GLUT4), LDH-A and Enolase (ENO1) (Fig. 7E). PDK1 inhibits the pyruvate dehydrogenase complex, PDH, preventing the utilization of acetyl-CoA into the TCA cycle for OXPHOS, while enhancing its reduction to lactate. To directly test the relevance of this pathway, we explored the activity of the PDK1 and glycolytic inhibitor dichloroacetate (DCA). As shown in Fig. 7F, DCA treatment of the *Slc25a1*^*-/-*^ MEFs rescued the rates of OCR, to levels nearly comparable to wild-type MEFs. Similar results were obtained by supplementing cells with the cell permeable, NAD^+^ precursor nicotinamide mononucleotide, NMN, which also partially rescued the rates of OXPHOS (Fig. 7G) and enhanced the NAD^+/^NADH ratio (Supplementary Fig. 6A).

Albeit we cannot exclude that additional mechanisms do exist, these data importantly demonstrate that the glycolytic switch is a driver of mitochondrial dysfunction and imply that the respiratory deficit conditions of SLC25A1 dysfunction, can at least partially be corrected.

**Fig. 8:**
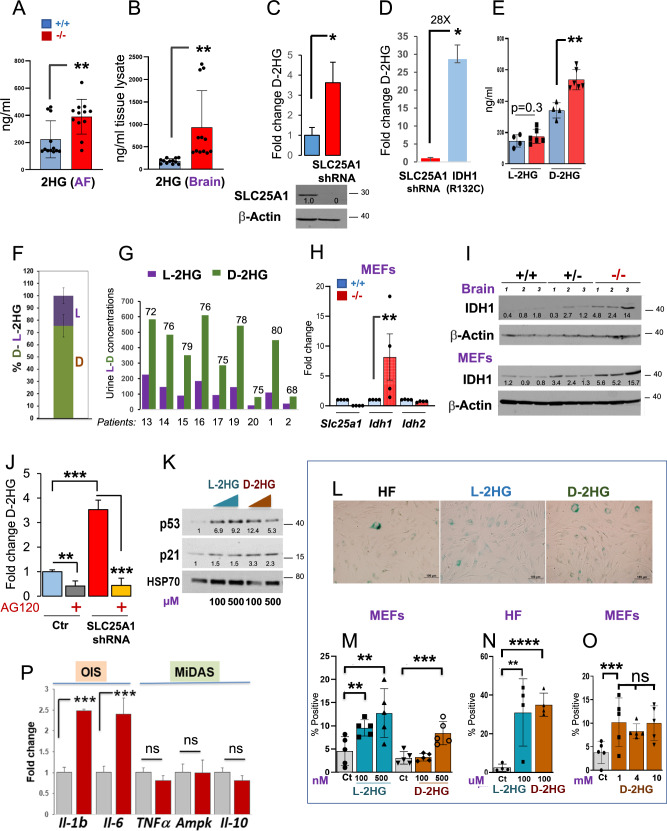
2HG induces an OIS-like pathway of senescence. 2HG enrichment in the amniotic fluid (**A**) and brain samples (**B**) obtained from *Slc25a1*^+/+^ (blue) and *Slc25a1*^-/-^ (red) embryos at E18.5 dpf (*n* = 4). **C** D-2HG enrichment in A549 cells harboring the shRNA mediated *SLC25A1* knock-down relative to control. **D** D-2HG enrichment in cells harboring the SLC25A1-KD or expressing the mutant IDH1^**R132C**^. **E** D or L-2HG enrichment in the amniotic fluids of *Slc25a1*^+/+^ (blue) and *Slc25a1*^-/-^ (red) embryos (n = 4-5). **F** Relative abundance of D- and L- 2HG (%) in the amniotic fluid samples of *Slc25a1*^-/-^ embryos compared to wild-type littermates (*n* = 2–3). **G** Urine concentration of D- and L- 2HG in patient samples. Data were extrapolated based on enrichment detected in two different studies. Patients 13-20 were from Ref. [11]; Patients 1 and 2 from Ref. [13]. mRNA (**H**) and protein (**I**) levels of SLC25A1, IDH1 and IDH2 in the indicated MEFs or brain samples (*n* = 3-4). **J** D-2HG enrichment in A549 cells harboring the SLC25A1-shRNA treated with AG120. Results were obtained from 3 experiments combined. **K** Expression levels of p53 and p21 in human fibroblast treated with the indicated concentrations of L- or D-2HG. Results are representative of two separate experiments. **L** Representative images of SA-β*-*GAL staining of control human fibroblast cells treated with L-or D- 2HG for 72 hours (Bar = 100 µM). Quantification of SA-β*-*GAL staining of MEFs cells (**M**, **O**) or control human fibroblasts (HF, **N**) treated with indicated concentrations of L-or D-2HG for 3 to 5 days (Bar = 100 µM). Bars represent SD from 2 separate experiments. **P** mRNA levels of the OIS (brown) and MiDAS (green) SASPs in cells treated with D-2HG. Data are from two experiments and are presented as mean value ± SD. In all blots, quantification was performed using ImageJ normalized to housekeeping protein (β-Actin or Hsp70), and results are presented as a fold change relative to control.

In cells lacking SLC25A1 low levels of D-2HG are produced by wild-type IDH1 and induce OIS-*lp*. We next sought to determine the characteristics of the 2HG produced in cells when SLC25A1 is deficient. In agreement with previous observations [8, 16], the total pool of 2HG was moderately elevated in the amniotic fluids of Slc25a1 deficient mice albeit being higher in the brains, reaching only a five-fold average enrichment compared to control (Fig. 8A, B). Similarly, the acute SLC25A1 knock-down (KD) led to a four-to-five-fold accumulation of D-2HG, again demonstrating that loss of SLC25A1 activity directly, but modestly induces enrichment of this metabolite (Fig. 8C). Consistent with this notion, the levels of D-2HG in fibrosarcoma cells, HT-1080, which harbor one of the most common *IDH1* mutations, IDH1^R132C^, were more than 30 fold higher compared to cells harboring the SLC25A1-KD (Fig. 8D). Given that different pathways exist for the synthesis of the D- or of the L-enantiomer, we employed a chiral derivatization method [40] to resolve the 2HG composition. With this approach, we established that the D-2HG represents the main pool enriched in *Slc25a1*^*-/-*^ MEFs and brains (Fig. 8E, F and not shown). A similar prevalence of D-2HG was also detected in nine patients affected by D/L-2HGA (previously published in Refs. [11, 13]) (Fig. 8G).

It is now emerging that wild-type IDH1/2 proteins can also produce D-2HG, albeit at much lower rates and concentrations compared to their mutant counterparts [16]. We found that the expression of IDH1, but not of IDH2, was increased in the *Slc25a1*^*-/-*^ MEFs, brains, as well as in the patient-derived fibroblasts, suggesting a role for this protein (Fig. 8H, I and Supplementary Fig. 6B, C, E). To explore the contribution of IDH1 to the accumulation of the D-2HG enantiomer, we employed the specific inhibitor, AG120, which was initially developed as an inhibitor for mutant IDH1, but more recently it was shown to have inhibitory activity at low concentrations against wild-type IDH1 in many cell lines [41]. As seen in Fig. 8J, in cells harboring the SLC25A1-KD, the accumulation of D-2HG was nearly entirely suppressed by AG120, but not by the IDH2 specific inhibitor AGI-6780 (Supplementary Fig. 6D). Further, similarly to AG120, the IDH1 shRNA led to a reduction of the D-2HG enantiomer in the patient derived cells (Supplementary Fig. 6E).

Given these results, we explored the idea that low levels of 2HG can induce p53-mediated senescence. To this end, we treated with low doses 2HG the MEFs or the primary human fibroblasts (HF) [34]. Low doses (nM or uM) of either D- or L-2HG strongly induced p53 and p21 (Fig. 8K) and the SAβ*-*GAL signal, indicative of senescence (Fig. 8L–N). This effect did not increase when the concentrations were titrated up to 1- to-10 mM (Fig. 8O), reflective of tumor concentrations, suggesting that induction of senescence is a mechanism by which normal cells respond to low levels of 2HG. Moreover, treatment with D-2HG induced SASPs typical of OIS, but not of MiDAS, particularly IL-1α and IL-6 (Fig.8P).

Thus, 2HG selectively induces the pro-inflammatory OIS arm of SASPs, but not the MiDAS-SASPs.

### Treatment of zebrafish embryos with 2HG partially recapitulates the phenotypes of SLC25A1 loss

**Fig. 9:**
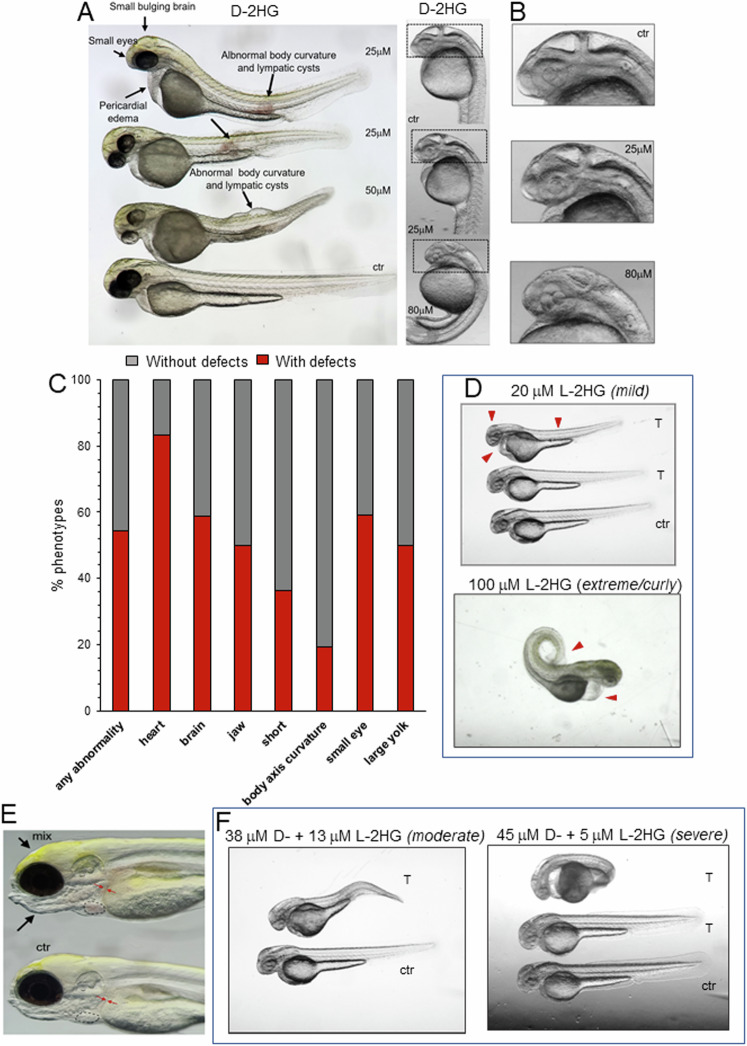
2HG perturbs embryonic development in zebrafish embryos. **A** Phenotypes of zebrafish embryos treated with 25 µM or 50 µM D-2HG at 2 dpf. Embryos were injected at blastula-stage. Prominent alterations are indicated by arrows, as small eyes, bulging brains, enlarged yolk sack, pericardial edema, altered body curvature, reduced body length, lymphatic cysts, and disorganized cellular structure of tissues, most prominently seen in the brain. **B** Phenotypes of 25 µM or 80 µM D-2HG treated embryos at 36 hpf. Top embryo is control. Higher magnification images of the head show the dose-dependent tissue disorganization of the brain. **C** Percent of zebrafish with or without defects untreated or treated with 2HG (control *n* = 4-15, 2HG treated *n* = 18-57). Data represent the results of 3 repetition experiments of embryos treated with different doses of 2HG. **D** Phenotype of embryos treated with the indicated doses of L-2HG at the indicated concentrations. Red arrows point to alterations in brain size, pericardial edema, enhanced body curvature and enlarged yolk. **E** Five days dpf Casper larvae. The top fish was treated with 30 µM D-2HG plus 20 µM L-2HG at 1 dpf. The lower fish is control. The treated larva has an enlarged heart (outlined with dashed line) and large diameter blood vessels (indicated with arrows). The treated larva additionally has a dysmorphic jaw and slightly bulging brain. **F** Treatment of embryos at 36 hpf, with the indicated mixtures using different ratios of D-2HG and L-2HG.

The effects of 2HG on embryonic development have only been studied directly in the two-cells to blastocyst transition in murine embryos [42]. Given the predominant abundance of D-2HG in D/L-2HGA sustained by SLC25A1 dysfunction, we explored its contribution to embryonic development by using the zebrafish model as an experimental platform. By performing dose-response experiments, we observed no overt toxicity at the concentrations used, and we detected relevant phenotypes at concentrations ranging between 20-100 μM. The main organ affected was the brain, which was small and bulging (Fig. 9A, B and quantified in 9C). Alterations in the vertebral column and in the body curvature were also significant. In addition, many treated embryos exhibited alterations in the size of the head, jaw and eyes, larger hearts as well as pericardial edema. At an 80 μM D-2HG dose, embryos exhibited prominent disorganization of the brain tissue (Fig. 9B). Because of the low abundance of L-2HG in D/L-2HGA and in cells lacking SLC25A1 observed previously, we also explored the effects of combining both enantiomers, at ratio typical of D/L-2HGA. As shown in Fig. 9D, low concentrations of L-2HG alone induced a mild phenotype, which became severe at higher doses. The combination D-2HG/L-2HG induced a cardiovascular phenotype consisting of heart enlargement and reduced ability of the heart to pump blood (Fig. 9E and Supplementary Video 1). Further, mixing D- and L-2HG at doses at which neither enantiomer alone induced severe alterations, worsened the phenotype (Fig. 9F), suggesting a pathogenic role for the presence of both enantiomers in SLC25A1-mediated D/L-2HGDH. However, all these phenotypes were less prominent relatively to complete depletion of *slc25a1* with the targeted morpholino, which resulted in relatively high percent of lethality, as we showed before [6]. We conclude that 2HG at least partially contributes to the embryonic developmental alterations seen in D/L-2HGA sustained by SLC25A1 deficiency.

### The OIS-*lp* and MiDAS pathways co-operate to restrict growth in a p53-dependent fashion

**Fig. 10:**
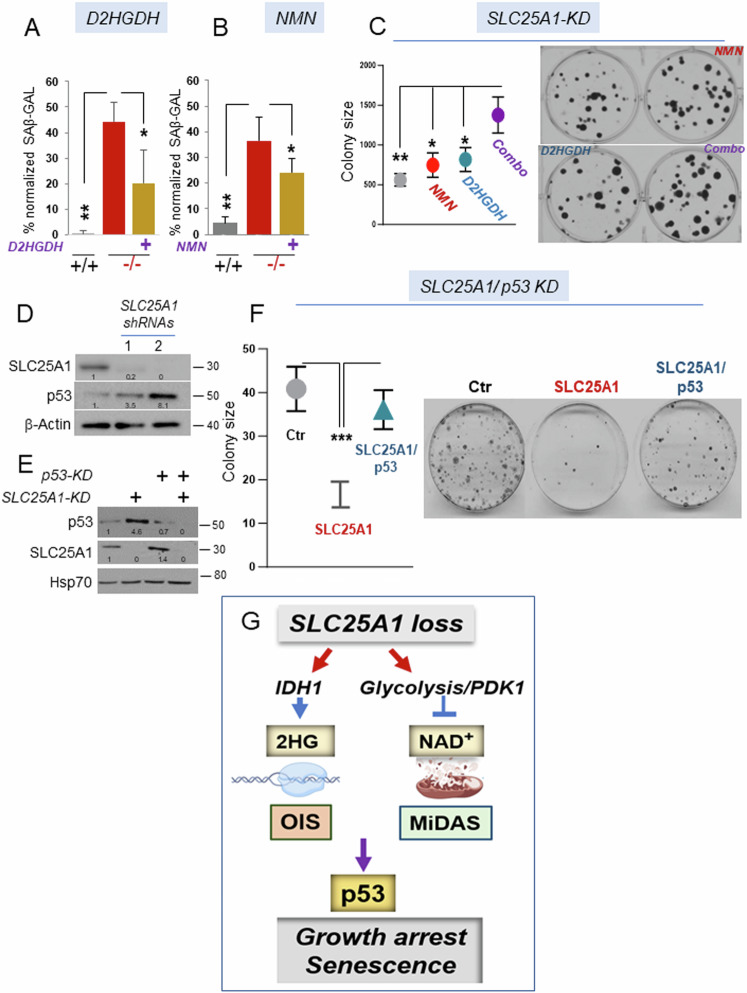
The growth defect imposed by SLC25A1 deficiency is p53-dependent. Quantification of the SAβ*-*GAL positive (%) MEF cells in the presence or absence of D2HGDH (**A**) or treated with NMN (**B**) and normalized to the total number of cells identified with DAPI counter-staining. **C** Quantification (with ImageJ) and representative images of colony formation assay of cells transduced with the lentivirus harboring the SLC25A1-shRNA (SLC25A1-KD) and treated with NMN, or over-expressing D2HGDH, alone or in combination (indicated as *combo*). **D** SLC25A1 and p53 expression levels in A549 cells transduced with the control lentivirus (pLKO, ctr) or with lentivirus vectors harboring two different SLC25A1 shRNAs (1 and 2). **E** Levels of expression of SLC25A1 and p53 in the double knock-down experiments performed in A549 cells. **F** Colony formation assay in A549 cells in the presence and absence of the SLC25A1-KD and p53-KD alone, or in combination. Quantification of colonies size was performed with ImageJ using multiple plates and bars represent SD. Unpaired non-parametric t test was used. **G** Model representing the effects of SLC25A1 loss (see also the discussion), and depicting the main pathways identified in this study leading to the induction of the *OIS-lp* and MiDAS programs. In all blots, quantification was performed using ImageJ normalized to housekeeping protein (β-Actin or Hsp70), and results are presented as a fold change relative to control.

The qualitative analysis of the SASP in the brains and in the *Slc25a1*^*-/-*^ MEFs, showed a mixed pattern of senescence involving pro-inflammatory signals derived from OIS, as well as SASPs typical of MiDAS. We have partially dissected these events as we have shown that 2HG is, per se, able to induce the inflammatory pro-oncogenic SASP arm, while enhanced glycolysis and decreased NAD^+^ drive mitochondrial dysfunction. To understand the significance of these senescence programs, we introduced in the *Slc25a1*^*-/-*^ MEFs the cDNA encoding for the D-2HGDH clearing enzyme, D-2HGDH. D-2HGDH significantly, but not completely reduced the extent of senescence (Fig. 10A and see Supplementary Fig. 7A for the expression levels of D-2HGDH). On the other hand, treating *Slc25a1*^*-/-*^ MEFs with either NMN (Fig. 10B) or with DCA (not shown), also partially reduced the proportion of senescent cells. To then determine the overall impact of these senescent programs on proliferation, we performed colony forming assays in cells harboring the *SLC25A1* knock-down. While treatment with NMN or over-expression of D-2HGDH alone modestly influenced colony forming ability, the combination had a more significant additive effect enhancing colony size and number (Fig.10C). Importantly, this cooperative effect was specific for cells harboring the SLC25A1-KD, as neither NMN nor D2HGDH over-expression enhanced proliferation rates in the absence of the SLC25A1-KD (Supplementary Fig. 7B, C). Hence, these two senescence programs act together to restrict growth in SLC25A1 deficient cells.

The OIS program serves as a fundamental tumor-suppressive mechanism, preventing cells that have acquired proliferative capacity from progressing through the cell cycle *via* the activation of tumor suppressors, including p53. Therefore, we sought to assess the relevance of p53 activity. First, we confirmed that the acute knockdown of SLC25A1 using two distinct shRNAs enhanced p53 expression (Fig. 10D), demonstrating that SLC25A1 depletion directly induces p53. To explore whether such induction is responsible for the proliferation defect of SLC25A1-deficient cells, we employed a controlled isogenic system, A549 cells, where the effects of the SLC25A1-KD were studied in the presence or absence of the p53-KD, again using colony forming assays. As shown in Fig. 10E, we were able to effectively achieve a double knock-down of both proteins. The results of these experiments clearly demonstrated that the proliferation defect due to SLC25A1 depletion could be alleviated by simultaneously knocking down p53, hence reinforcing the idea that p53 activity restrict the expansion of cells with dysfunctional SLC25A1 (Fig. 10F).

## Discussion

The pathogenesis of genetically inherited human diseases due to *SLC25A1* loss is still largely unclear. Our results provide evidence that its inactivation leads to the engagement of two pathways of senescence, OIS and MiDAS. They further show that accumulation of low doses 2HG and mitochondrial dysfunction driven by enhanced glycolysis and depletion of NAD^+^, respectively, trigger these senescent programs (summarized in Fig. 10G). We have also demonstrated that the molecular and phenotypic alterations arising from SLC25A1 deficiency are in part sustained by the accumulation of D-2HG, based on the observation that in zebrafish treatment with this enantiomer was able to recapitulate some salient phenotypic features that overlap with the *slc25a1* depletion induced by a specific morpholino. The main impact of these findings is in the observation that SLC25A1 loss leads to only a modest enrichment of 2HG, yet these low concentrations appear to be pathogenic. In addition, wild-type IDH1 is responsible for the synthesis of the D-enantiomer at least in the model systems that we have studied. Finally, D-2HG induces p53 and senescence, a finding only in apparent contrast with its definition of as “oncometabolite”.

The above observations have several implications. First, consistent with the RNAseq data showing an enrichment of pro-oncogenic pathways, an important question is whether SLC25A1 loss is eventually oncogenic. It is relevant to note that as in the case of VCFS and DGS, there is a link between 2HG-acidurias and cancer risk. In fact, children affected by 2HG-acidurias sustained by mutations of *IDH1/2* or *D/L-2HGDH*, exhibit high cancer incidence, particularly of oligodendriomas, low grade gliomas, anaplastic astrocytomas and medulloblastomas [43–48]. Second, it is well known that the presence of proto-oncogenes in normal cells induces the activity of the p53 tumor suppressor to elicit senescence as a mechanism to halt the proliferative capacity of cells that have acquired oncogenic potential. Our data now reveal that 2HG induces a p53/p21-mediated OIS like pattern of senescence and thus, imply that p53 interacts with potentially oncogenic metabolic products similarly to its interaction with oncogenic proteins. This is a novel concept that will offer a paradigmatic example of how alterations in the metabolism may lead to senescence and cancer. Third, low levels of enrichment of 2HG are currently considered non-pathogenic, but this moiety is present in modest amount in metabolic disorders and in tumors that do not harbor IDH1/2 mutations [15]. Our results challenge this concept, revealing multifaceted mechanisms of action of 2HG depending upon the cellular context, and as such, will likely drive further investigations into the broader pathogenic role of this metabolite as well as of wild-type *IDH1/2* genes in human diseases.

The mechanisms by which SLC25A1 loss leads to deterioration of OXPHOS were up to this point unclear, but we have now identified the depletion of NAD^+^ and high glycolytic rates, presumably due to the HIF1α-mediated response, as at least in part responsible for this effect in the cellular models relevant to human diseases that we have examined. Noticeably, an NAD^+^-driven pseudohypoxia response has been linked to aging [25], and in the seminal work of the Campisi’s laboratory MiDAS was detected in murine models of progeroid syndromes [24]. *Slc25a1* homozygous embryos exhibit growth retardation, wrinkled skin, and facial deformities including anophtalmia, which are seen in models of progeria [49]. Interestingly, deficiencies of other mitochondrial transporters of the SLC25 family are known to lead to mitochondrial dysfunction, early aging and progeroid features [50, 51]. Together with our findings, these observations raise the possibility that the MiDAS response in the context of SLC25A1 deficiency leads to an accelerated aging progeroid phenotype. The identification of drugs (DCA, NMN) able to reverse this deficit provides novel means of therapeutic interventions. Lastly, we have observed an enhancement of citrate-dependent pathways and lipid build-up in *Slc25a1* deficient embryos. Citrate and lipids are well known mediators of inflammation and of senescence [52], suggesting that a maladaptive compensatory dysmetabolic response to replenish the cytosolic pool of citrate and lipids also contributes to the loss of proliferative capacity.

In summary, data presented in this work reveal that complete elimination of SLC25A1-mediated signaling during embryonic development leads to molecular and phenotypic alterations more complex than anticipated and that disorders sustained by its loss should be reclassified as dysmetabolic and mitochondrial disorders. We predict that with the identification of strategies that correct the molecular alterations due to SLC25A1 dysfunction, this study will inform on therapeutic strategies able to ameliorate at least some aspects of these disorders.

## Supplementary information

Supplementary Figures and Methods

Supplementary Video 1

Uncropped autoradiograms

## Data Availability

All data will be made available on public databases and upon request.
