# Supplementary material for: Inactivation of the SLC25A1 gene during embryogenesis induces a unique senescence program controlled by p53
Source: Cell Death Differ. 2024 Dec 29;32(5):818–36. doi: 10.1038/s41418-024-01428-w (PMC12089371; doi:10.1038/s41418-024-01428-w)
Supplement: Supplementary file 1 — Supplementary Figures and Methods [file 41418_2024_1428_MOESM1_ESM.pdf]

SUPPLEMENTARY INFORMATION

Inactivation of the *SLC25A1* gene during embryogenesis induces a unique senescence program controlled by p53

Anna Kasprzyk-Pawelec[1], Mingjun Tan[1,2], Raneen Rahhal[1,] Alec McIntosh[1], Harvey R. Fernandez[1,3], Rami Mosaoa[1,4], Lei Jiang[5], Gray W. Pearson[1], Eric Glasgow[1], Jerry Vockley[6], Christopher Albanese[1], and Maria Laura Avantaggiati[1,#]

[1] Georgetown University Medical Center, Lombardi Comprehensive Cancer Center, Washington, D.C., USA

[2] Penn State College of Medicine, Department of Cellular & Molecular Physiology, Hershey, PA, USA

[3] University of Virginia Medical Center, Charlottesville, VA, USA.

[4] Department of Biochemistry, Faculty of Science, King Abdulaziz University, Jeddah, Saudi Arabia

[5] Department of Molecular and Cellular Endocrinology, Arthur Riggs Diabetes and Metabolism Research Institute, City of Hope Medical Center, Comprehensive Cancer Center, City of Hope Medical Center, Duarte, CA, USA

[6] Department of Pediatrics, Division of Genetic and Genomic Medicine, UPMC Children's Hospital of Pittsburgh, Pittsburgh, PA, USA

Correspondence:

Maria Laura Avantaggiati: ma364@georgetown.edu

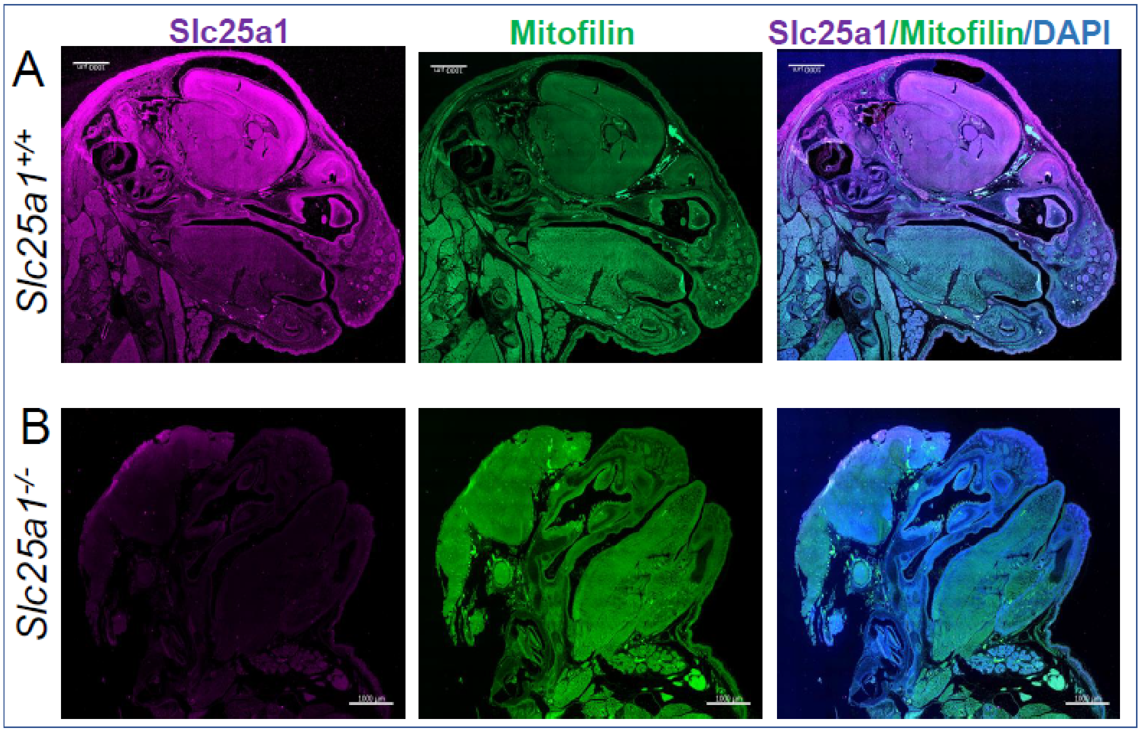

Figure S1: Expression of SLC25A1 protein in the craniofacial region.

A Staining with anti-SLC25A1, anti-mitofilin and DAPI in embryos at E19 dpf. Bar=1000 μm.

B Staining as in A, in E19 dpf *Slc25a1* nullizygous embryos.

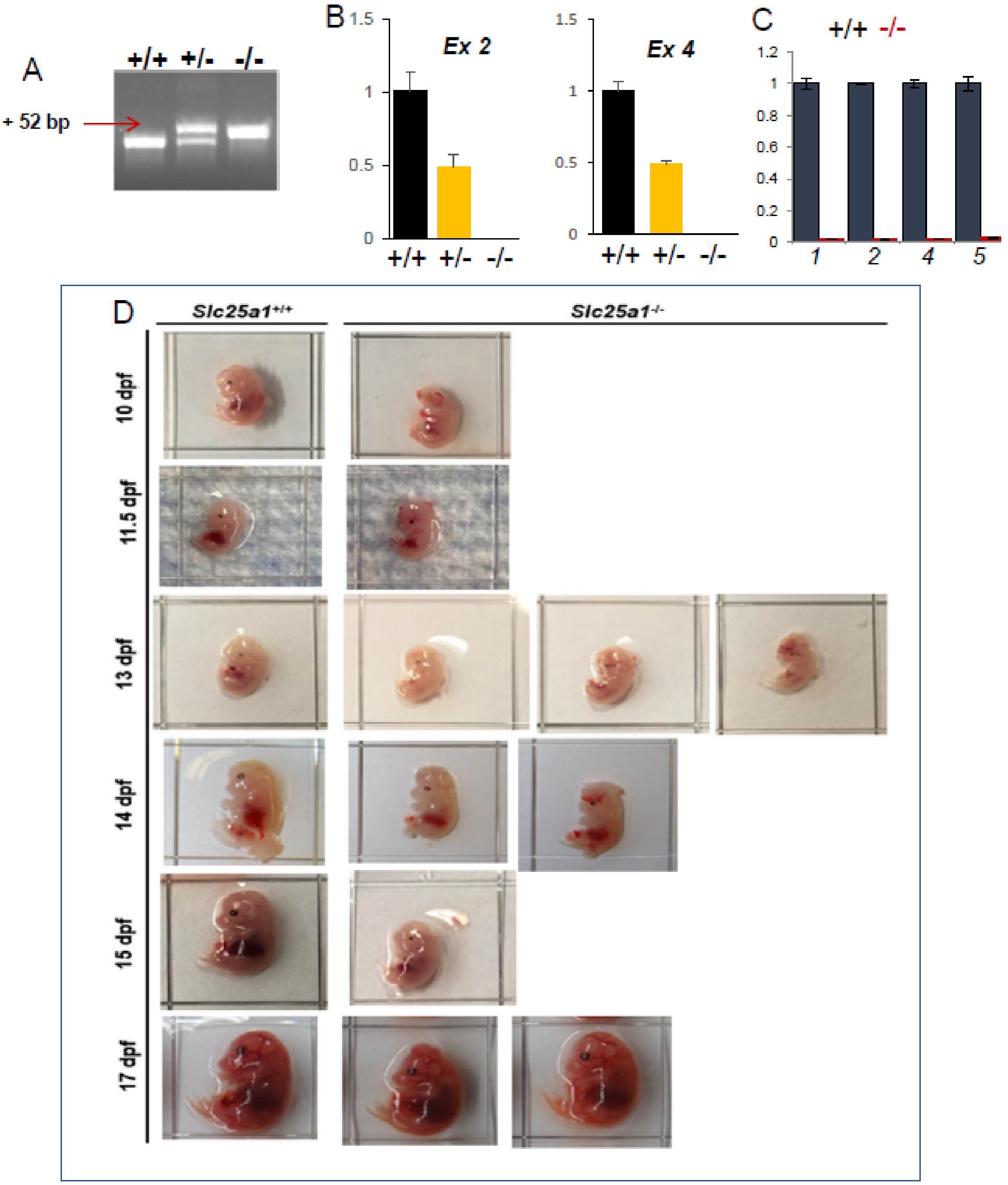

Figure S2: SLC25A1 deficient embryos can be recovered at all stages of embryonic development.

A Genotyping strategy for the *Slc25a1*$^{+/-}$ and *Slc25a1*$^{-/-}$ mice. The insertion of the *LoxP* site between *exons* 4 and 5 in chromosome 16 creates a difference of 52 bp that allows distinguishing of the +/+; +/-; and -/- mice (see Ref. 27 for illustration of the trapping cassette).

B,C Quantitative rtPCR with primers spanning *exons 2* and *4* of the Slc25a1 transcript in the brain (**B**) and *exons 1, 2, 4, 5* in MEFs (**C**).

D Representative images of *Slc25a1*$^{+/+}$ and *Slc25a1*$^{-/-}$ embryos collected at the indicated stages of embryonic development. Identical scale in each set.

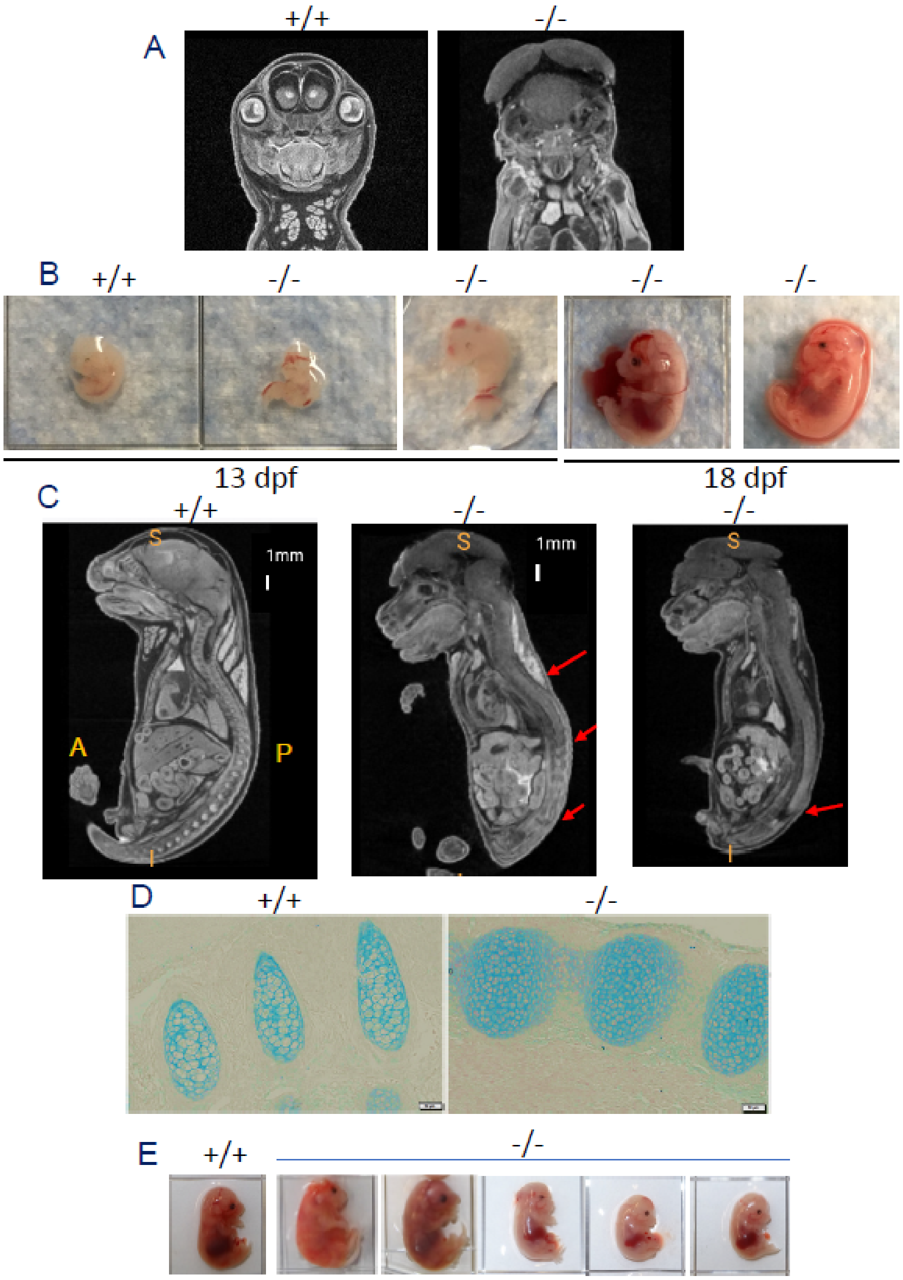

Figure S3: Heterogeneity of the phenotypes induced by Slc25a1 loss.

A Facial dysmorphic features of *Slc25a1*$^{-/-}$ embryos at E18.5 dpf compared to wild-type and detected with CT scan (UC Davis library).

B Hemorrhagic phenotype in *Slc25a1*$^{-/-}$ embryos affecting the brain, liver and abdominal organs.

C-D Alterations of the vertebral column in *Slc25a1*$^{-/-}$ embryos at E18.5 dpf, including abnormal curvature, increased thickness and poor separation of the vertebrae (**C**) and alcian blue staining of cartilage in the vertebral column (**D**).

E Phenotype of the five *Slc25a1*$^{-/-}$ embryos used for the metabolomic studies. One representative wild-type embryo is also shown.

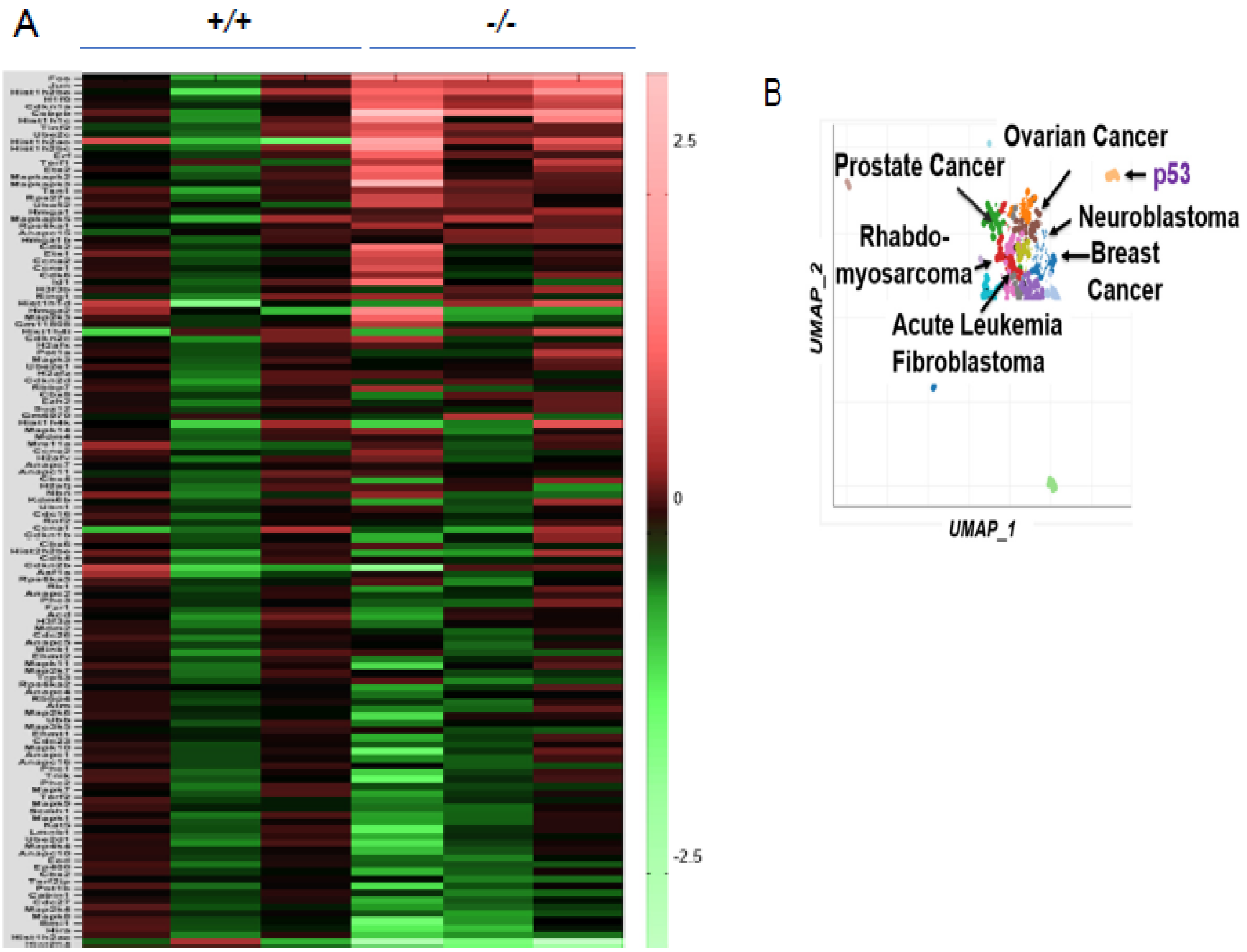

Figure S4: Transcriptomic analysis of *Slc25a1*$^{-/-}$ embryos.

A Heatmap of upregulated and downregulated genes related to senescence. The GSEA enrichment set containing genes related to senescence and with a log2 fold changes were plotted in prism graphpad to generate the heatmap.

B Transcriptomic data derived from each of the embryos were used for a Hue-Saturation-Value (HSV) analysis. The Enrichr tool was employed to identify gene sets that are significantly more active in E-SEV and moderately elevated in E-MOD *versus* wild-type (*WT.***>***.E-MD.***>>***.E-SV);* $p < 0.05$.

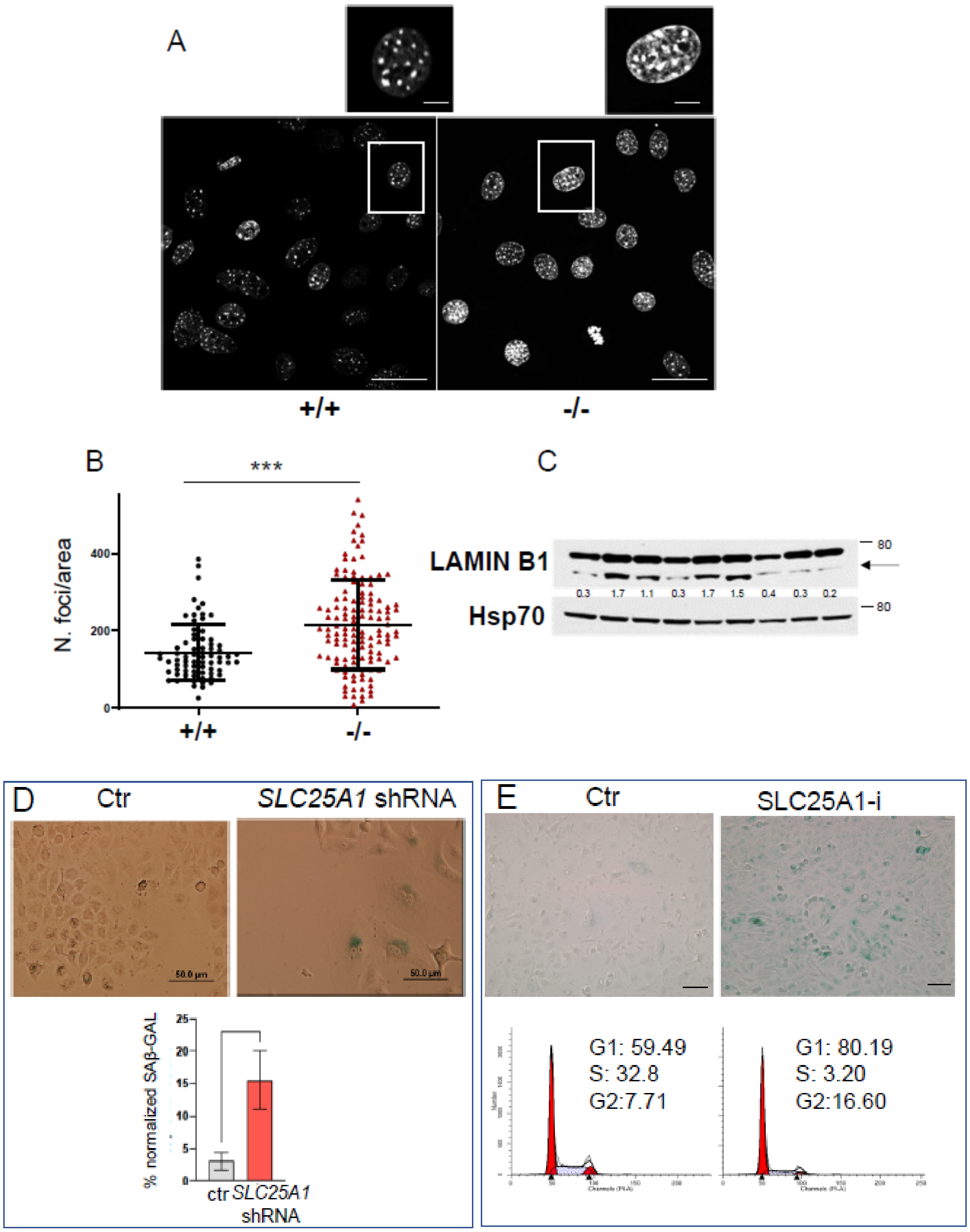

Figure S5: Induction of senescence in cells with dysfunctional SLC25A1.

A DAPI staining of MEFs derived from wild-type and Slc25a1$^{-/-}$ embryos showing senescence-associated heterochromatic foci.

B Quantification of nuclear foci per area obtained with the count particles function of the ImageJ software from multiple fields (5-6) each containing 3-10 cells.

C Immunoblot of the indicated cells with the monoclonal antibody for Lamin B1 (indicated by the arrow).

D A549 cells were transduced with the control lentivirus or with lentivirus harboring the SLC25A1 specific shRNA (see Fig.10 for SLC25A1 levels), selected with puromycin for 5 days and stained with β-GAL.

E SA-β-GAL activity and cell cycle analysis of A549 cells treated with the SLC25A1-i.

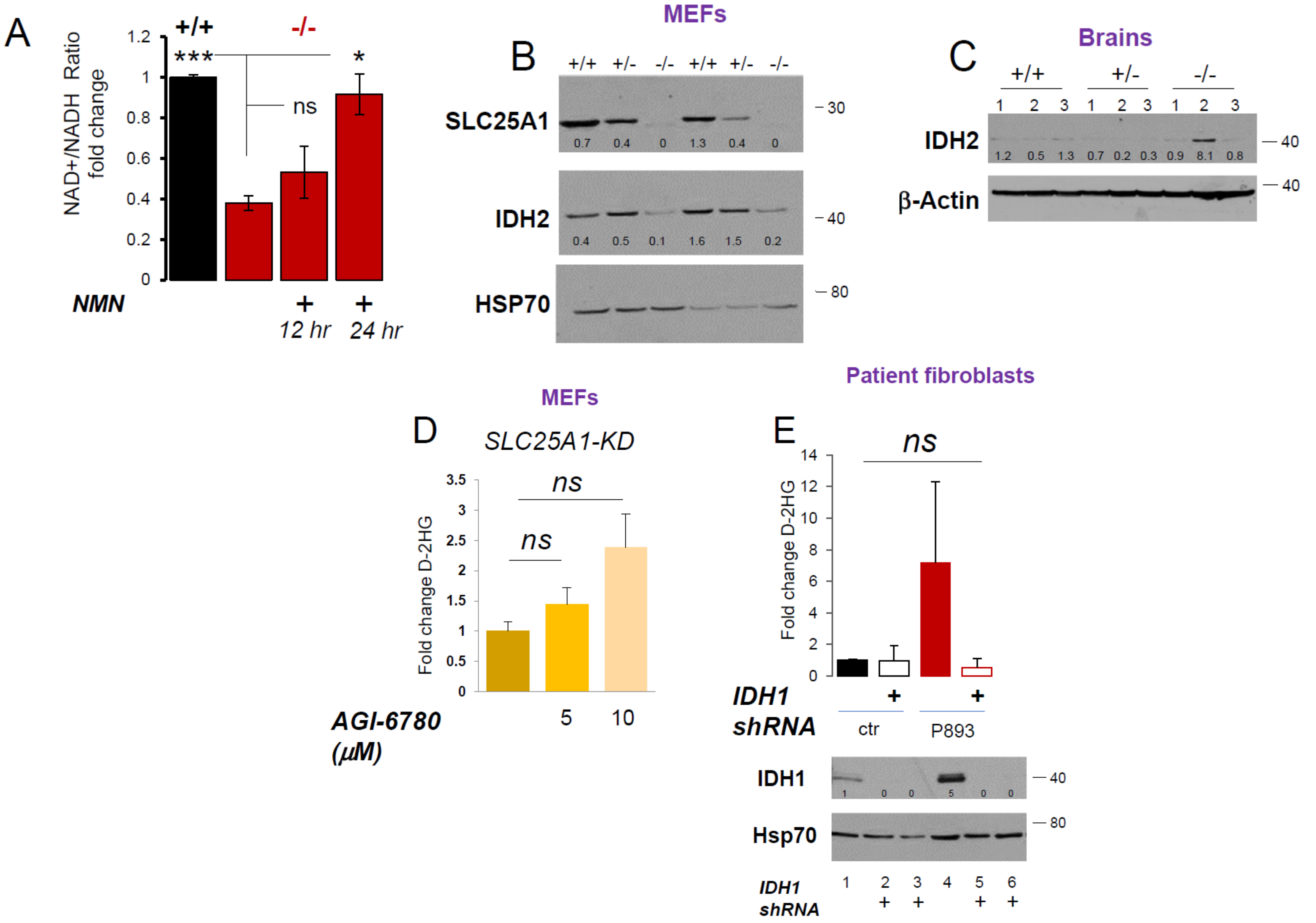

Figure S6: Characterization of the NAD+/NADH ratio and of D-2HG in SLC25A1 deficient cells.

A Quantification of the NAD+/NADH ratio in wild-type (black bar) and Slc25a1$^{-/-}$ (red bars) MEFs in the absence of presence of treatment with NMN. Cells were treated with 50 μM NMN for 12 or 24 h, as indicated. Results were pooled from two separate experiments. SD=standard deviations.

B-C Expression levels of IDH2 and SLC25A1 in the MEFs (**B**) and brains (**C**) of the indicated mice. **D** Levels of enrichment of D-2HG in cells treated with the IDH2 inhibitor AGI-6780.

E Levels of enrichment of D-2HG (top panel) and IDH1 expression (bottom panel) in control fibroblasts or in the patient derived P893 fibroblasts transduced with control lentivirus or with the lentivirus harboring the *IDH1*-specific shRNAs. Cells were collected at 48 hours (lanes 2 and 5) and at 5 days (lanes 3 and 6) after antibiotic selection. Note that none of the differences in this panel were statistically significant, likely due to the difficulty of measuring low D-2HG levels in the small percentage of transfected cells in these assays.

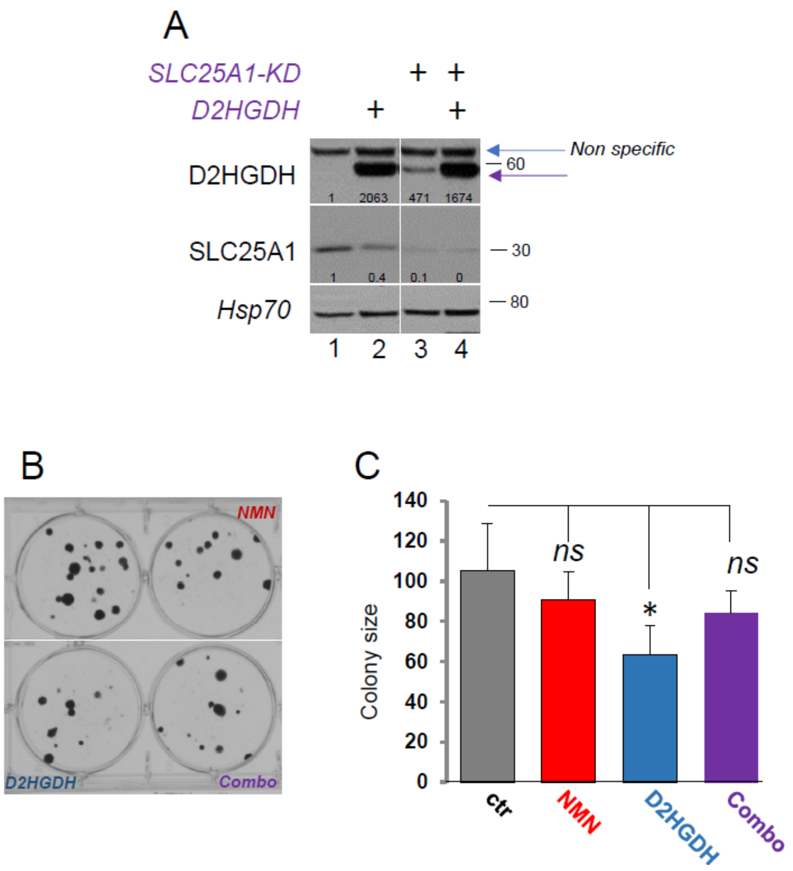

Figure S7: Effects of D2HGDH over-expression.

A Immuno-blots of H1299 cells over-expressing D2HGDH (lanes 2 and 4) in the presence or absence of the SLC25A1-KD (lanes 3,4). The levels of D2HGDH, SLC25A1 and Hsp70 are indicated. Arrows point to a non-specific band detected by the D2HGDH antibody. Images were taken from the same autoradiogram, at identical exposure and non-relevant lanes were cut in the middle.

B,C Representative images (**B**) and quantification (**C**) of colony forming assays in cells transduced with control lentivirus and treated with NMN or expressing D2HGDH, alone or in combination (related to Fig. 10A-C). In all blots, quantification was performed using ImageJ normalized to housekeeping protein (β-Actin or Hsp70), and results are presented as a fold change relative to control.

Supplementary Video 1. Heart dysfunction in zebrafish embryos treated with 40 μM D-2HG at 36 hpf.

The top embryo is treated with D-2HG, the bottom is control.

Table S1. Murine primers used for real-time PCR

Genes	Forward primer (5'→3')	Reverse primer (5'→3')
Acc1	GGACCACTGCATGGAATGTTAA	TGAGTGACTGCCGAAACATCTC
Fasn	GGAGGTTGCTTGGAAGAG	CTGGATGTGATCGAATGCT
Tnf	CCCTCACACTCAGATCATCTTCT	GCTACGACGTGGGCTACAG
Idh1	ATGCAAGGAGATGAAATGACACG	GCATCACGATTCTCTATGCCTAA
Idh2	GGAGAAGCCGGTAGTGGAGAT	GGTCTGGTCACGGTTTGGAA
Ampk	CAGGCCATAAAGTGGCAGTTA	AAAAGTCTGTCGGAGTGCTGA
Il-1b	GCAACTGTTCCTGAACTCAACT	ATCTTTTGGGGTCCGTCAACT
Il-6	TAGTCCTTCCTACCCCAATTTCC	TTGGTCCTTAGCCACTCCTTC
Il-10	GCTCTTACTGACTGGCATGAG	CGCAGCTCTAGGAGCATGTG
Ppia	CAGTGCTCAGAGCTCGAAAGT	CACCGTGTTCTTCGACATCA
Actb	ATGGAGGGGAATACAGCCC	TTCTTTGCAGCTCCTTCGTT
Tbp	GGGGTCATAGGAGTCATTGG	CATCTCAGCAACCCACACAG
Slc25a1 EXON2	GAAATCTGCATCACCTTCCCG	GTGGGTTCGCTCGTTCATCT
Slc25a1 EXON4	TCGAGTTCCTCAGCAACCAC	CCATAGGGCACACGACTACC
Slc25a1 GENOTYPING	TCTGTTGAAACGACCCGGAG	AGGTCTCCATTCTCCAGGCT

Supplementary Methods

Genotyping

Mouse tail biopsies and agarose gel electrophoresis was used for analysis of PCR products. The PCR conditions used were as follows: 94 °C for 1 minute, 5 cycles at 94 °C for 30 s, 63 °C for 40s, 68 °C for 1 min; followed by 30 cycles (94 °C for 30 seconds, 55 °C for 40 seconds, and 72 °C for 1 min), 72 °C for 7 min then kept in 4 °C before use. The primers used for genotyping are indicated in Table S1.

Cell culture

The A549 and H1299 cell line were obtained from the tissue culture core facility at LCCC. Cells were grown in Dulbecco's Modified Eagle's Medium (DMEM 4500 mg/L glucose) supplemented with 10% fetal bovine serum, 4 mM L-glutamine, 1 mM sodium pyruvate and 1% antibiotic solution. The S*LC25A1*-specific shRNA vectors were purchased from Sigma (TRCN0000232825; TRCN0000255350) and validated before (6,18,27). Octyl-2-HG (Cayman 16366, 16367) was diluted in PBS pH 7.2. NMN was purchased form Selleckchem (S5259) and DCA from Sigma (347795).

Mouse embryonic fibroblasts (MEFs) isolation

Freshly dissected embryos at E10-E14 were used for isolation of mouse embryonic fibroblasts (MEFs). Brain, liver, internal organs and tail were removed, and the remaining tissues were rinsed with PBS and transferred to a 10 cm plate. Embryos were then minced with a sterile razor blade in the presence of TrypLE™ Express Enzyme (Gibco™, 12604021) following by 10 min incubation on a shaker at RT. Finally, the cells were pipetted multiple times to further disintegrate the tissue, and then the freshly isolated cell were cultured in high glucose DMEM medium (Dulbecco's Modified Eagle Medium with 10% fetal bovine serum, 4500 mg/L glucose, 4 mM L-glutamine, 1 mM sodium pyruvate and 1% antibiotic solution).

Growth assays and Colony formation assay

H1299 and p53H1299 (or indicated cells) were plated in a six-well plate at density 500 cells/well for 14 days, and the medium was replaced every 3 days. Additionally, indicates cells were treated with NMN 24h after plating for 13 days, and the medium was replaced every 3 days. Cells were fixed in 4% paraformaldehyde,

stained with crystal violet and imaged/photographed to visualize the results. Quantification was done by manual counting and measuring stained area with Image J.

Protein analysis and immunoblot

Proteins from cells or frozen tissue samples, grinded in liquid nitrogen, were homogenized in RIPA buffer supplemented with cOmplete Mini Protease Inhibitor Tablets (Roche, 11836153001). Protein quantification was done by using Coomassie (Bradford) Protein Assay Kit (Pierce). Equal amount of protein lysate was loaded and separated in an SDS-polyacrylamide gel electrophoresis using Novex™ 4-20% Tris-Glycine Mini Gel (Invitrogen, XP04200PK2) and transferred to PVDF membrane (Millipore, IPVH304F0). The membrane was blocked in blocking buffer with 10% horse serum to prevent non-specific binding, followed by incubation with different antibodies: SLC25A1 (Santa Cruz, sc-86392 or Proteintech, 15235-1-AP), ACC1 (Cell Signaling, #3676), FASN (Cell Signaling, #3180), IDH1 (Proteintech, 12332-1-AP), AMPK-α (Cell Signaling, #5832), pAMPK-α (Cell Signaling, #2535), AMPK-α (Cell Signaling, #5832), Chk1 (Cell Signaling, Cat#2360), HIF1-alpha (Novus Biological, Cat#NB100-105), p21 Waf1/Cip1 (Cell Signaling, #37543), mTOR (Cell Signaling, #2983), pH2A.X (Cell Signaling, #9718), H2A.X (Cell Signaling, #7631), Lamin B1 (Cell Signaling, #17416), p53 (Santa Cruz, sc-126 and Sigma Aldrich, OP29-100UG), D2HGDH (Proteintech, 13895-1-AP). Appropriate horseradish peroxidase–conjugated secondary antibody (Invitrogen) was applied after incubation with primary antibody, SuperSignal™ West Pico PLUS Chemiluminescent Substrate (Thermo Scientific, 34577) or SuperSignal™ West Dura Extended Duration Substrate (Thermo Scientific, 34075) were used for protein detection. β-Actin (Santa Cruz, sc-47778) and HSP70 (Santa Cruz, sc-24) served as a loading control.

Embryos, brains and amniotic fluids collection

Pregnant mice were euthanized at different stages of pregnancy and whole embryos were collected. Typically, embryos were harvested at E9.5-E19.5, and tips of tails were collected for genotyping. For IHC, embryos were fixed in 4% paraformaldehyde overnight, transferred to 80% EtOH and sent for histological processing. Paraffin embedding, sectioning and hematoxylin/eosin staining of embryos tissue sections were performed by Histology and Microscopy Core of Georgetown University or by VitroVivo Biotech. To collect the amniotic fluid, and avoid cross-contamination, we typically selected embryos distally located. After transferring the embryos to a clean dish, the amniotic fluid was collected by 1-mL syringe and 27-gauge needle. For biochemical and molecular

analysis, indicated organs were dissected, quickly washed in PBS and snap frozen in liquid nitrogen. For the collection of the brains, skin and skull (when present) were carefully removed. The isolated brains were quickly washed in PBS twice, snap frozen in liquid nitrogen and stored at -80°C until processed for further analyses.

CT Images acquisition from the International Mouse Phenotyping Consortium.

The IMPC has provided CT images of *Slc25a1*$^{-/-}$ mice without description of the phenotypes. Analysis of these images and phenotype identification was performed in house at GUMC through the help of the Preclinical Imaging Research Laboratory (PIRL) facility.

2HG quantification with LC/MS/MS and GS/MS/MS

For LC/MS a targeted metabolomics method was used to quantitate 2-HG using QTRAP® 7500 LC-MS/MS System (Sciex, MA, USA). The 2HG standard was from Sigma Aldrich (# 90790-10MG). For the purpose, 75 µL of extraction buffer (methanol/water 50/50) containing 200 ng/mL of 4-nitrobenzoic acid as internal standard for negative mode was added to the cell pellet and sample tube was plunged into dry ice for 30 sec and 37 °C water bath for 90 sec. This cycle was repeated for two more times and then samples were sonicated for 1 minute. The samples were vortexed for 1 min and kept on ice for 20 minutes followed by addition of 75 µL of ACN. The samples were incubated at -20 °C for 20 minutes for protein precipitation. The samples were centrifuged at 13,000 rpm for 20 minutes at 4 °C. The supernatant was transferred to MS vial for LC-MS analysis. 20 µL of each prepared sample was mixed to generate the pooled QC sample. For GC-TOF amniotic fluids or tissue samples were processed using 500 µL of 50% methanol in water containing the internal standard (4-Nitrobenzoic acid, prepared in MeOH at a concentration of 20 µg/mL). The samples were homogenized on ice to ensure tissue lysis and metabolite extraction was then vortexed for 2 minutes. A volume of 10 µL was withdrawn from the homogenized tissue suspension for the protein quantification assays. The samples were vortexed for 2 minutes and left for 2 hours at room temperature. The BCA kit used for the protein quantification assay was Pierce™ BCA Protein Assay Kit (Cat # 23225). A volume of 20 µL of methoxyamine (20 mg/mL) was added to the dried tissue extracts and then heated in an agitator at 60 °C for 30 minutes. This was followed by 100 µL of MSTFA. The vials were transferred to an agitator to heat at 60 °C for 30 more minutes. Finally, the vials were capped and a volume of 1.5 µL was injected directly to the GC-MS. The samples were allowed to react at room temperature for 20 minutes before being transferred to the

GC for injection. Briefly after the derivatization process, a volume of 1.5 µL of the derivatized solution was injected in (1:10) split mode into an Agilent 7890B GC system (Santa Clara, CA, USA) that was coupled with a Pegasus HT TOF-MS (LECO Corporation, St. Joseph, MI, USA). Separation was achieved on a Rtx-5 w/Integra-Guard capillary column (30 m x 0.25 mm ID, 0.25 µm film thickness; Restek Corporation, Bellefonte, PA, USA), with helium as the carrier gas at a constant flow rate of 0.9 mL/min. The temperature of injection, transfer interface, and ion source were set to 150, 270, and 320 °C, respectively. The GC temperature programming was set to 0.2 minutes of isothermal heating at 70 °C, followed by 6 °C/min oven temperature ramping to 270 °C, a 7.0 minute isothermal heating of 270 °C, 20 °C/min to 320 °C, and a 2.0 min. isothermal heating of 320 °C. Electron impact ionization (70 eV) at full scan mode (40–600 *m*/*z*) was used, with an acquisition rate of 20 spectra per second in the TOF/MS setting.

Metabolomic and Lipidomics

Metabolomic (targeted and untargeted was performed as described before (Tan et al, 2020). The lipidomic method used in the brains of *Slc25a1*$^{-/-}$ mice, is designed to measure 19 classes of lipid molecules which includes diacylglycerols (DAG), chloesterol esters (CE), sphingomyelins (SM), phosphtatidylchloine (PC), triacylglycerols (TAG), free fatty acids (FFA), ceramides (CE), dihydroceramides (DCER), hexosylceramide (HCER), lactosylceramide (LCER), phosphatidylethanolamine (PE), lysophosphtatidylchloine (LPC), lysophosphatidylethnolamine (LPE),phosphatidic acid (PA), lysophosphatidic acid (LPA), phosphatidylinositol (PI), lysophosphotidylinositol (LPI), phosphatidylglycerol (PG) and phosphatidylserine (PS) using QTRAP® 5500 LC-MS/MS System (Sciex). For the purpose, 20 µL of each amniotic fluid sample was dissolved in 100 µL of chilled isopropanol containing internal standards was added and samples were vortexed. The samples were vortexed again for 1 min and kept on ice for 30 minutes. Samples were incubated at -20 °C for 2 hours for complete protein precipitation. The samples were centrifuged at 13,000 rpm for 20 minutes at 4 °C. The supernatant was transferred to MS vial for LC-MS analysis. Five µL of each sample was injected onto a Xbridge amide 3.5 µm, 4.6 X 100 mm (waters) using SIL-30 AC auto sampler (Shimazdu) connected with a high flow LC-30AD solvent delivery unit (Shimazdu) and CBM-20A communication bus module (Shimazdu) online with QTRAP 5500 (Sciex, MA, USA) operating in positive and negative ion mode. Obtained results were analyzed using MetaboAnalyst.

Brain TCA cycle analyses

Tissue samples were mixed with 400 µL of MeOH containing the internal standard (4-Nitrobenzoic acid) which was prepared in MeOH at a concentration of 30 µg/mL and homogenized for 60 seconds. A volume of 10 µL was withdrawn from the tissue suspension for the protein concentration assay. The samples were mixed for 2 minutes, left at -20 °C for 2 hours then centrifuged for 30 min at a speed of 16,000 X g and a temperature of 4 °C. The supernatant was separated and transferred to the GC vials then dried under vacuum at room temperature. Samples were derivatized and injected into GCMS instrument. Protein concentration was determined using BCA assay. The Briefly after the derivatization process, a volume of 2.0 µL of the derivatized solution was injected in a splitless mode into an Agilent 7890B GC system (Santa Clara, CA, USA) that was coupled with a Pegasus HT TOF-MS (LECO Corporation, St. Joseph, MI, USA). Separation was achieved on a Rtx-5 w/Integra-Guard capillary column (30 m x 0.25 mm ID, 0.25 µm film thickness; Restek Corporation, Bellefonte, PA, USA), with helium as the carrier gas at a constant flow rate of 0.8 mL/min. The temperature of injection, transfer interface, and ion source was set to 150, 270, and 320 °C, respectively. The GC temperature programming was set to 0.2 min. of isothermal heating at 70 °C, followed by 6 °C/min oven temperature ramping to 300 °C, a 1.0 min. isothermal heating of 300 °C, 20 ° C/min to 320 °C, and a 2.0 min. isothermal heating of 320 °C. Electron impact ionization (70 eV) at full scan mode (40–600 m/z) was used, with an acquisition rate of 20 spectra per second in the TOF/MS setting.

Quantitative real-time PCR

Total RNA was extracted from frozen tissue and cells pellet samples using TRIzol™ Reagent (Invitrogen, 15596026), followed by RNA quantification with NanoDrop (Implen, Munich, Germany). After genomic DNA treatment by DNase I (Invitrogen, AM2222), total RNA was used in cDNA synthesis with SuperScript™ IV Reverse Transcriptase (Invitrogen, 18090200) and random hexamers according to the manufacturer's instructions. The quantitative Reverse Transcriptase-PCR (qRT-PCR) was performed on the QuantStudio™ 12K Flex Real-Time PCR System (Applied Biosystems) with PowerUp™ SYBR Green Master Mix (Applied Biosystems). The obtained results were normalized to housekeeping genes and the relative gene expression fold changes were calculated using $2^{-\Delta\Delta CT}$ method. Primer sequences are provided in Table S1.

RNA sequencing

Brains isolated from 18.5 dpf embryos were used for RNA sequencing. Briefly, RNA isolatation and RNA sequencing (RNAseq) was performed by Novogene. Raw intensity data was background corrected and filtered of low expression genes across samples (low expression genes across samples (low expression genes across samples (<100 read) prior to analysis. Normalization and differential expression were performed in R using the DESeq2 package. A cutoff of $p < 0.05$ and $|FC| > 2$ were used to determine differentially expressed genes. The GSEA Java-based software package from The Broad Institute was used to identify top enriched pathways in *Slc25a1*$^{+/+}$ and *Slc25a1*$^{-/-}$ brains. Reference genome and gene model annotation files were downloaded from genome website directly. Index of the reference genome was built using Hisat2 v2.0.5 and paired-end clean reads were aligned to the reference genome using Hisat2 v2.0.5. feature Counts v1.5.0-p3 was used to count the reads numbers mapped to each gene. FPKM of each gene was calculated based on the length of the gene and reads count mapped to this gene. FPKM, expected number of Fragments Per Kilobase of transcript sequence per Millions base pairs sequenced, considers the effect of sequencing depth and gene length for the reads count at the same time, and is currently the most commonly used method for estimating gene expression levels. Differential expression analysis was performed using the DESeq2 R package (1.20.0). The P values were adjusted using the Benjamini & Hochberg method. Corrected P-value of 0.05 and absolute fold change of 2 were set as the threshold for significantly differential expression.

Immunohistochemistry and immunofluorescence

Age-matched embryos were removed from the uteri and fixed in 4% paraformaldehyde in phosphate-buffered saline and paraffin embedded, sectioned in the mid sagittal plane and stained with hematoxylin and eosin (H&E) and anti-Slc25a1 antibody (Proteintech, 15235-1-AP), used at 1/150 dilution. Consecutive sections with the primary antibody omitted were used as negative controls. Stained slides were scanned using the VS120 Virtual Slide Microscope (Olympus). For immunofluorescence (IF) staining, FFPE embryo sections were deparaffinized and sodium citrate was used for antigen retrieval. Slides were washed in PBS, blocked using TBST+20% Aquablock, and incubated with primary antibodies: anti-Slc25a1 (Proteintech, 15235-1-AP) and p21 (Santa Cruz, sc-756). Finally, samples were washed in PBST and incubated with appropriate secondary

antibody and DAPI or Hoechst (Sigma-Aldrich). IF samples were acquired using a Zeiss LSM800 laser scanning confocal microscope and analyzed using ImageJ/Fiji software.

Electron microscopy

Pregnant females were euthanized and perfused with 2.5% glutaraldehyde and 2% PFA in 0.15M cacodylate buffer. Following osmication, dehydration, and embedding in Epon 814, embryos heads were sectioned transversely (at the midpoint) at 120 nm using an ultramicrotome. Ultrathin sections were placed in silicon wafers and carbon taped in aluminum stubs for scanning electron microscopy (SEM) imaging in a Helios NanoLab 660 dual beam microscope. To maximize the collection of the backscattered electrons and produced TEM-like images, a high contrast solid-state backscatter electron detector, in magnetic immersion mode and 4 μm working distance, was used. The acquisition was performed using 2 kV and 0.40 nA as the current landing. Overview images including the entire section were taken at a magnification of 5,000X and analyzed using ImageJ software.

Senescence-associated β-Gal staining

Cells grown on a 6-well plate were washed with PBS, fixed with fixative solution and stained with x-gal according to the manufacturer protocol (Cell Signaling, #9860). After counterstaining with DAPI, cells were imaged using an Olympus IX-71 Inverted Epifluorescence Scope and multiple images of each well were taken for quantification.

Lentivirus production and infection

Control empty vector and vector shIDH1 were purchased from Sigma. Lentiviruses were produced in Lenti-X 293T cells with Third Generation Packaging Mix (Applied Biological Materials Inc., Cat#LV053) according to the manufacturer's instruction. Cells were transduced with lentiviruses and Polybrene Infection/Transfection Reagent (Millipore) reagents for 24 hrs and 0.5 μg/ml puromycin was used to obtain stably expressing cell lines.

D2HGDH overexpression (Plasmids and transfection)

D2HGDH expression plasmid (OriGene, RC207367) and pRc/CMV expression plasmid (Invitrogen) were used. Cells were plated in a six-well plates and allowed to grow overnight, and transfected using Lipofectamine 2000 (Invitrogen, cat. 11668-030) and OPTI-MEM reduced serum media (Gibco, cat. 31985-

062). The medium was replaced after 24 h. The D2HGDH overexpression was verified by Western blotting using the D2HGDH antibody (Proteintech, 13895-1-AP).

NAD/NADH measurement

Indicated MEF cells were collected, homogenized using extraction buffer II and filtered through a 10kD spin column (Abcam, ab93349). Measurements were performed using NAD/NADH assay kit (Abcam, ab65348) followed by assay kit protocol.

Seahorse extracellular flux analyzer measurements

Cells were seeded at 10 000 cells/well density in high glucose DMEM with 10% FBS, 1 mM pyruvate and 1% antibiotic solution, and the following day treated with the indicated drug/vehicle for 24 h. Seahorse measurements were done according to the manufacturer's instructions. Briefly: for the mitochondrial stress test, the medium was replaced with DMEM without FBS or bicarbonate, containing 5 or 10 mM glucose, 1 mM pyruvate, 2 mM glutamine and placed in a CO2 free incubator at 37 °C for 1 h, transferred to the Xf96 extracellular flux analyzer (Agilent). The program consisted of three measurements of OCR/ECAR before the injection of each drug: oligomycin (0.5 μM final concentration), FCCP (2 μM) and rotenone/antimycin (0.5 μM of each).

ETC Respiratory Complex activities. The activities of the ETC were assessed with the Seahorse analyzer with injections to study individual complexes as follows. For complex I after mild permeabilization, injection 1: Pyruvate 10mM, Malate 5mM; injection 2: Rotenone. For complex II: injection 1: Rotenone (to inhibit complex I), injection 2: Succinate (10mM), Injection 3: Atpenin A5 (complex II inhibitor). For complex III: injection 1: pyruvate 10mM, injection 2: antimycin. For complex IV: injection 1: pyruvate 10mM injection 2: sodium Azide (200uM). ATP synthase: injection 1: pyruvate 10mM, injection 2: oligomycin.

2HG analysis

The α-Hydroxyglutaric Acid was obtained from Cayman Chemicals. Diacetyl-L-tartaric anhydride (DATAN) was purchased from FisherScientific. All other reagents and solvents were of analytical and LCMS grade. Sample preparation: a volume of 50 μL from amniotic fluid was extracted with 250 μL of methanol containing 0.004 mmol/L of the internal standard (IS). Cell pellets were extracted with a volume of 1 mL of methanol containing 0.004 mmol/L of the internal standard. Samples were centrifuged for 20 minutes at 14,

000 X g and 4 °C. The supernatant was separated in a clean set of vials. The vial contents were evaporated to dryness at 22 °C by a gentle flow of nitrogen. The di-acetyltartaryl derivative was prepared by treating the dry residue with 50 µL of freshly made 50 mg/mL DATAN in Acetonitrile–acetic acid (4:1 by volume). The vials were capped and heated at 75 °C for 60 minutes. After the vial were cooled to room temperature, the mixture was evaporated to dryness by a nitrogen stream at room temperature, the residue was redissolved in 100 µL of distilled water, and 10 µL of the aqueous solution was injected on the LC column and analyzed by LCMS. The sample, prepared in the first step, were analyzed on a Sciex QTRAP 4500 mass spectrometer equipped with a Shimadzu Prominence UFLC XR System whereas, it was separated Phenomenex Lux 3 µm AMP, LC Column 150 x 3.0 mm, PN: 00F-4751-Y0. The column temperature was kept at 22 0C. Solvent A was 100% LCMS grade water with 0.1% formic acid and solvent B was 100% LCMS grade acetonitrile with 0.1% formic acid. The source temperature was kept at kept at 220 0C. The ion spray voltage was -4500 volts. The flow rate was consent throughout the method and was set to 0.3 mL/min. The mass spectrometry method was set to a Negative MRM mode with a cycle time of 0.99 second. The targeted masses of the derivatized 2-HG were m/z 363, 147, and 129 Da. The Entrance Potential (EP) was set to -10 volts and the Declustering Potential (DP), the Collision Cell Exit Potential (CXP) were set to values optimized previously for each assigned transition. The concentration of the analyte in urine was calculated by interpolation of the observed analyte/IS peak-area ratio based on the linear regression line for the calibration curve, which was obtained by plotting peak-area ratios vs analyte concentration. Data was analyzed using Analyst 1.7 and Sciex OS software for quantification.

D/L-2HG derivatization method

The α-Hydroxyglutaric Acid was obtained from Cayman Chemicals. Diacetyl-L-tartaric anhydride (DATAN) was purchased from FisherScientific. All other reagents and solvents were of analytical and LCMS grade. A volume of 50 µL from amniotic fluid was extracted with 250 µL of methanol containing 0.004 mmol/L of the internal standard (IS). Cell pellets were extracted with a volume of 1 mL of methanol containing 0.004 mmol/L of the internal standard. Samples were centrifuged for 20 minutes at 14, 000 X g and 4 °C. The supernatant was separated in a clean set of vials. The vial contents were evaporated to dryness at 22 °C by a gentle flow of nitrogen. The di-acetyltartaryl derivative was prepared by treating the dry residue with 50 µL of freshly

made 50 mg/mL DATAN in Acetonitrile–acetic acid (4:1 by volume). The vials were capped and heated at 75 °C for 60 minutes. After the vial were cooled to room temperature, the mixture was evaporated to dryness by a nitrogen stream at room temperature, the residue was redissolved in 100 µL of distilled water, and 10 µL of the aqueous solution was injected on the LC column and analyzed by LCMS. The sample, prepared in the first step, were analyzed on a Sciex QTRAP 4500 mass spectrometer equipped with a Shimadzu Prominence UFLC XR System whereas, it was separated Phenomenex Lux 3 µm AMP, LC Column 150 x 3.0 mm, PN: 00F-4751-Y0. The column temperature was kept at 22 0C. Solvent A was 100% LCMS grade water with 0.1% formic acid and solvent B was 100% LCMS grade acetonitrile with 0.1% formic acid. The source temperature was kept at kept at 220 0C. The ion spray voltage was -4500 volts. The flow rate was consent throughout the method and was set to 0.3 mL/min. The mass spectrometry method was set to a Negative MRM mode with a cycle time of 0.99 second. The targeted masses of the derivatized 2-HG were m/z 363, 147, and 129 Da. The Entrance Potential (EP) was set to -10 volts and the Declustering Potential (DP), the Collision Cell Exit Potential (CXP) were set to values optimized previously for each and every assigned transition. The concentration of the analyte in urine was calculated by interpolation of the observed analyte/IS peak-area ratio based on the linear regression line for the calibration curve, which was obtained by plotting peak-area ratios vs analyte concentration. Data was analyzed using Analyst 1.7 and Sciex OS software for quantification.

Zebrafish

All zebrafish procedures were performed in accordance with NIH guidelines for the maintenance and use of laboratory animals and approved by the Georgetown University Institutional Animal Care and Use Committee Protocol #2017-0078. Mixed wild-type, or *mitfa*$^{w2/w2}$; *mpv17* $^{a9/a9}$ (casper), embryos were obtained by pair-wise and group breeding, and raised in fish water (0.3 g/L sea salts) at 28°C. For drug treatments, (2R)-Octyl-α-hydroxyglutarate (referred to as D-2HG) (Cayman Chemicals) and (2S)-Octyl-α-hydroxyglutarate (referred to as L-2HG) (Cayman Chemicals), solutions were prepared in fish water. Embryos were treated at blastula, 1 dpf, or 2 dpf stages at a density of 100ul drug solution per embryo. Chorions were removed at 1 dpf by 10 min incubation in 200ug/ml pronase. Drug solutions were replaced daily.

Statistical analyses

For the analysis of the RNAseq experiments in the brain, differentially Expressed Genes were obtained using DESeq2. Significantly upregulated Genes (FDR < 0.05) were assessed for pathway enrichment using the Reactome 2022 database via EnrichR. Significantly enriched Pathways were clustered via GeneSetCluster using a distance score that reflects similarities in genes mapping to multiple significantly enriched pathways. Similar processes were summarized by secondary annotation. The p-values of Reactome pathway enrichment were displayed as a Heatmap via GraphPad Prism. For all experiments, results are presented as mean value ±SD or SEM, unless specified otherwise. Statistical significance was assessed using unpaired non parametric t-test, unless indicated otherwise. Significant differences were graphed using GraphPad Prism software and $p < 0.05$ was considered as statistically significant.
