# Supplementary material for: Inactivation of the SLC25A1 gene during embryogenesis induces a unique senescence program controlled by p53
Source: Cell Death Differ. 2024 Dec 29;32(5):818–36. doi: 10.1038/s41418-024-01428-w (PMC12089371; doi:10.1038/s41418-024-01428-w)

Figure 1B

HSP70 embryo 1

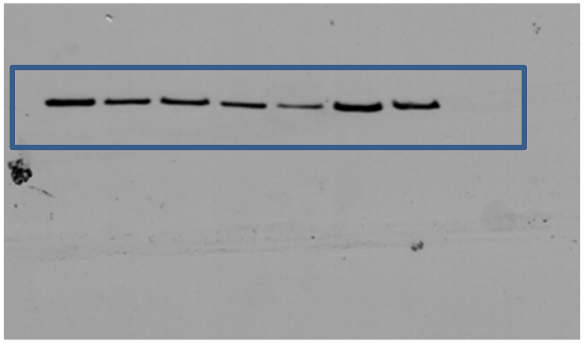

HSP70 embryo 2

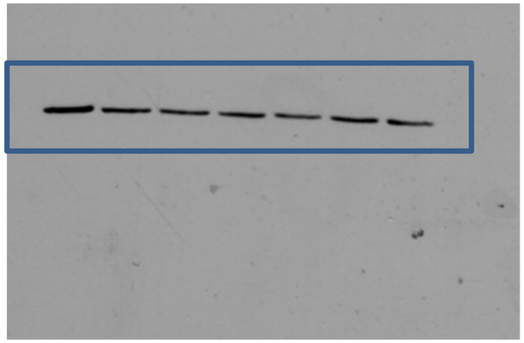

SLC25A1 embryo 1

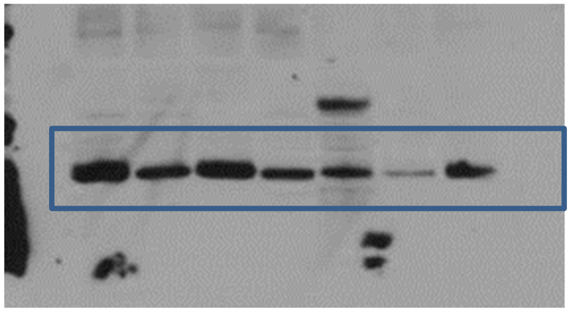

SLC25A1 embryo 2

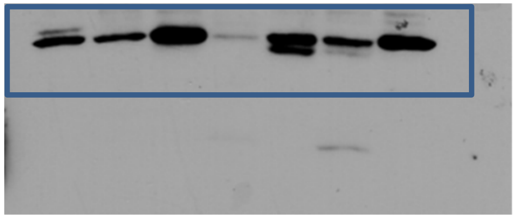

Figure 1E

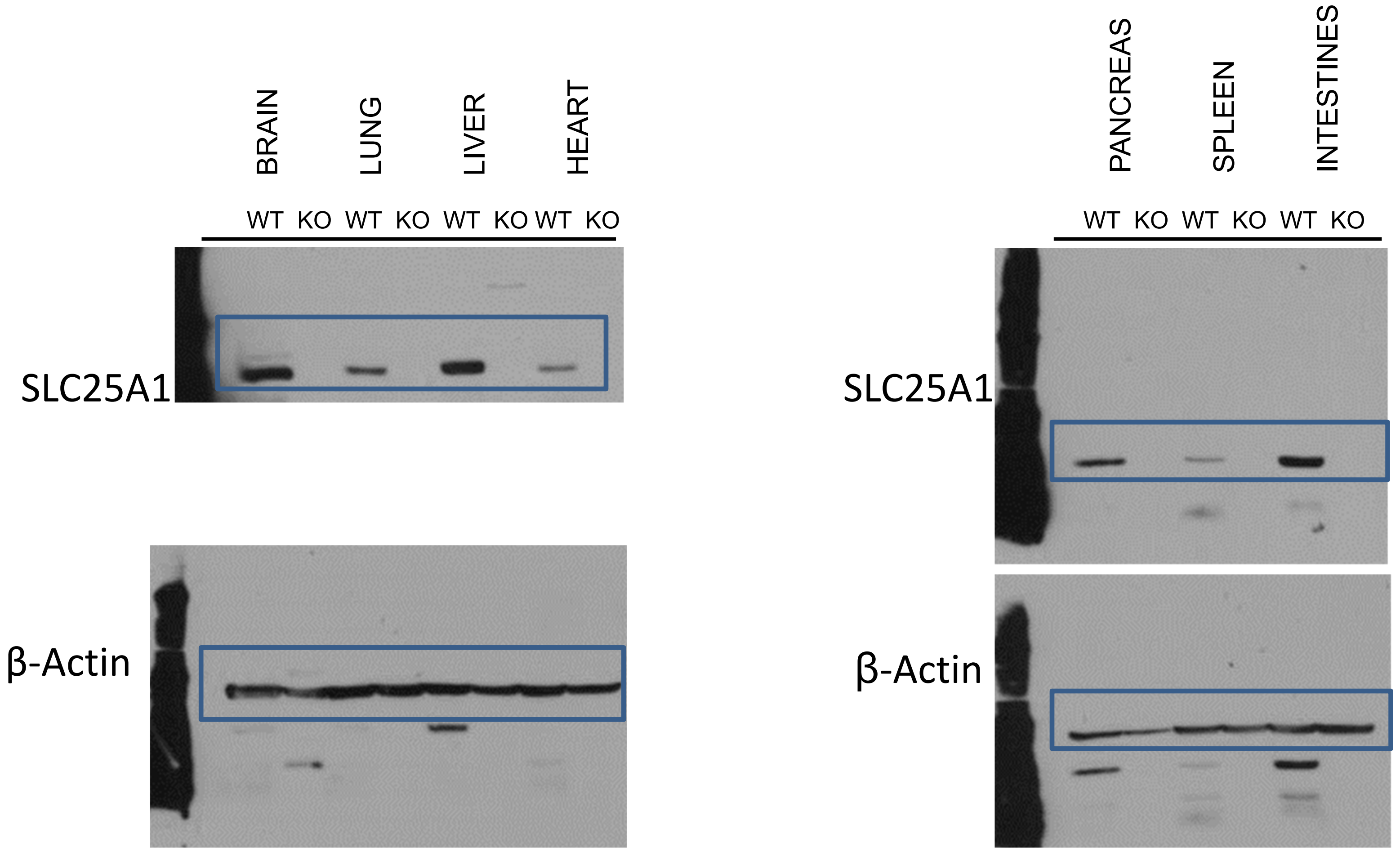
BRAIN
LUNG
LIVER
HEART
WT KO WT KO WT KO WT KO
SLC25A1
β-Actin
PANCREAS
SPLEEN
INTESTINES
WT KO WT KO WT KO
SLC25A1
β-Actin

Figure 3A FASN

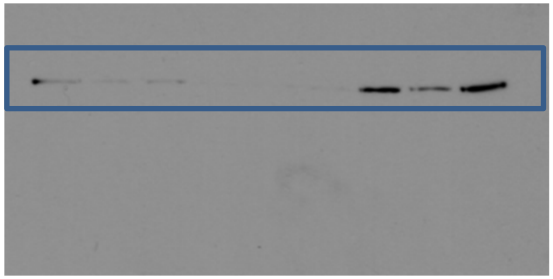

Figure 3A ACC1

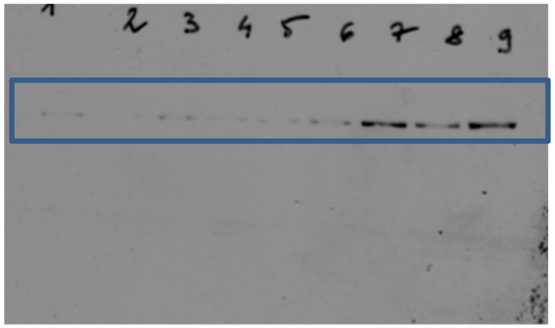

Figure 3A SLC25A1

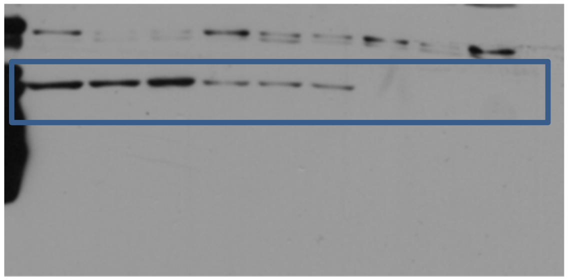

Figure 3A β-Actin

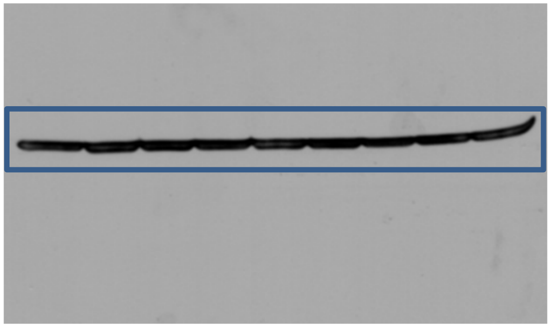

Figure 3A β-Actin

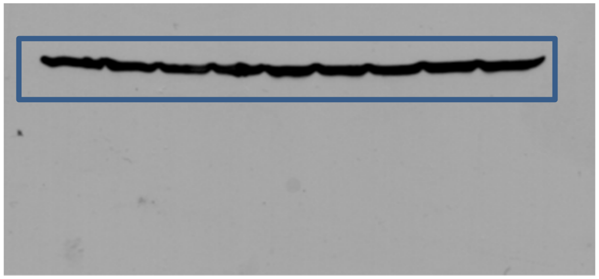

Figure 3A HSP70

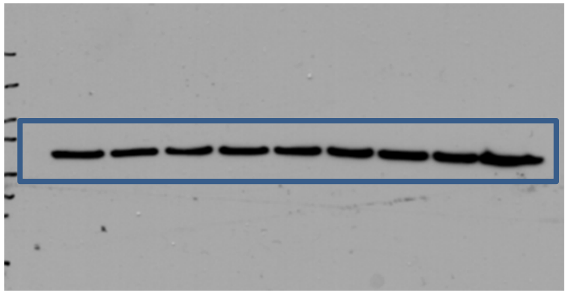

Figure 3B FASN

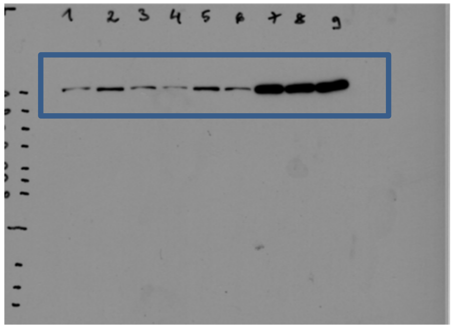

Figure 3B ACC1

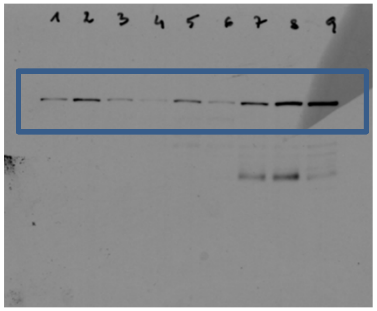

Figure 3B SLC25A1

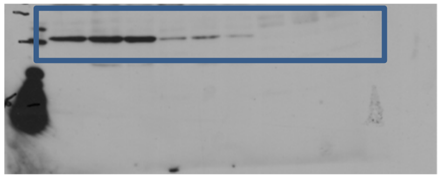

Figure 3B β-Actin

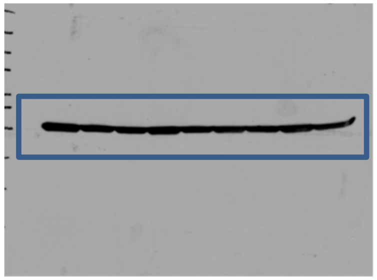

Figure 3B β-Actin

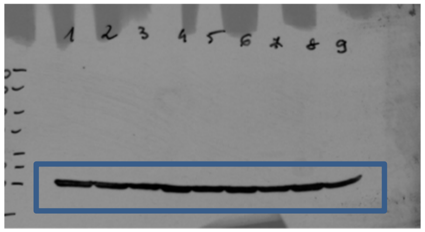

Figure 6C pAMPK

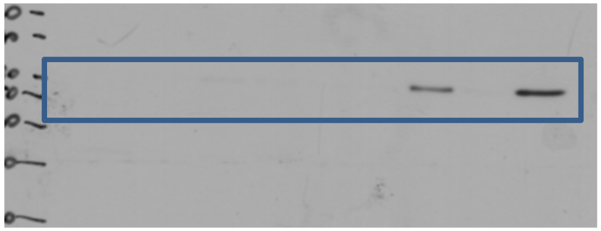

Figure 6C CHK1

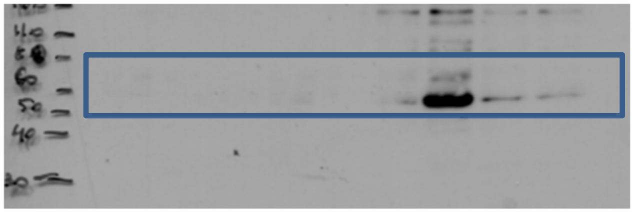

Figure 6C β-Actin

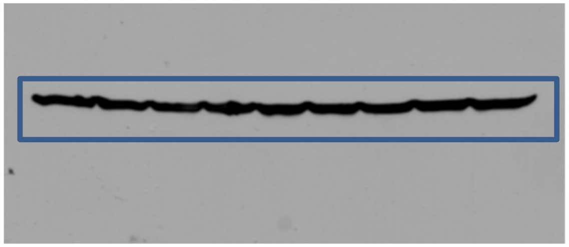

Figure 6C pH2AX

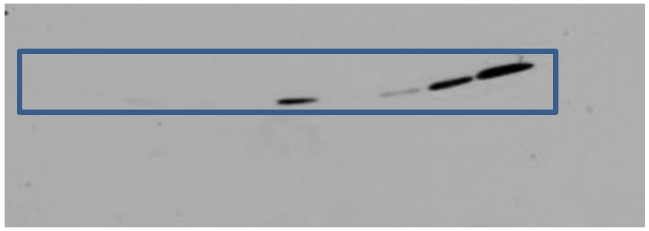

Figure 6C p21

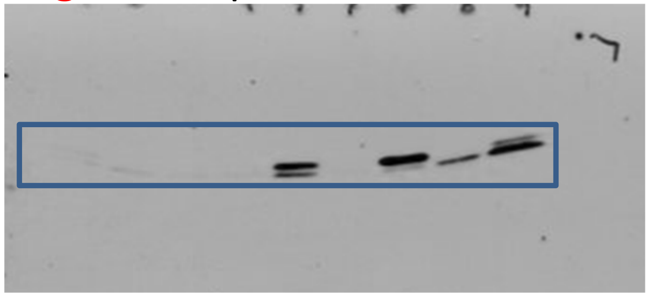

Figure 6C HSP70

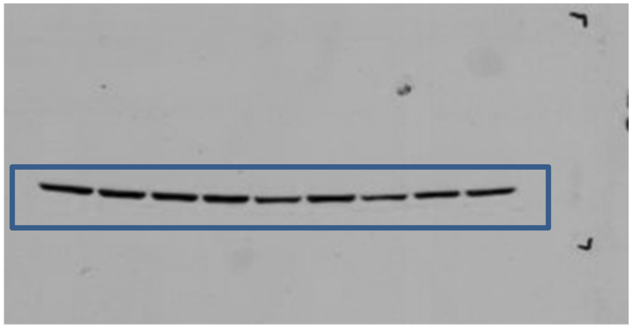

Figure 6C H2AX

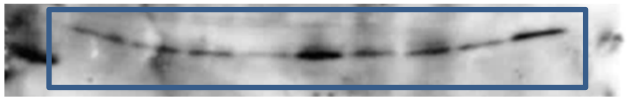

Figure 6C HIF1α

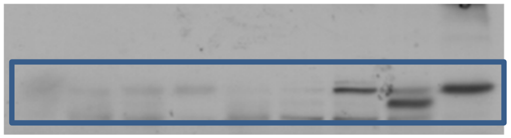

Figure 6C β-Actin

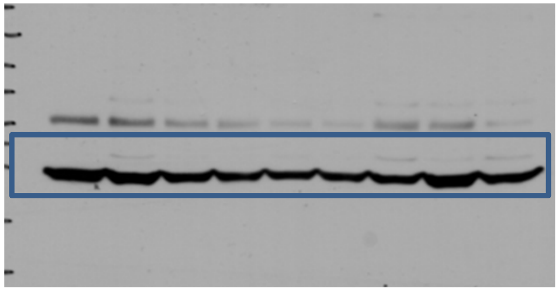

Figure 6C AMPK

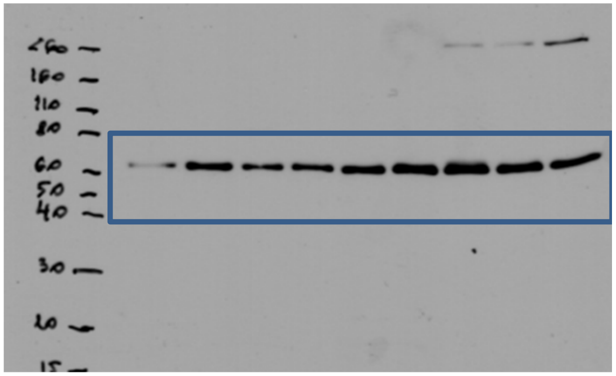

Figure 6C β-Actin

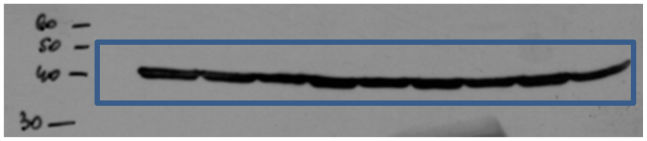

Figure 6D p53

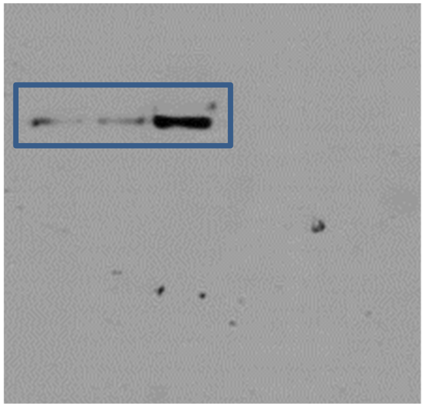

Figure 6D p21

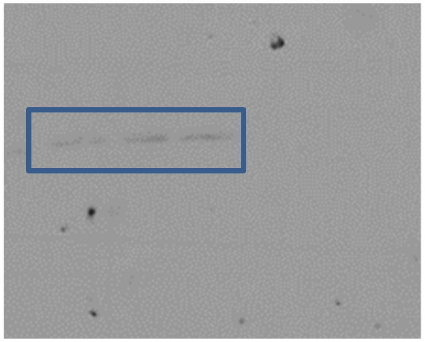

Figure 6D HSP70

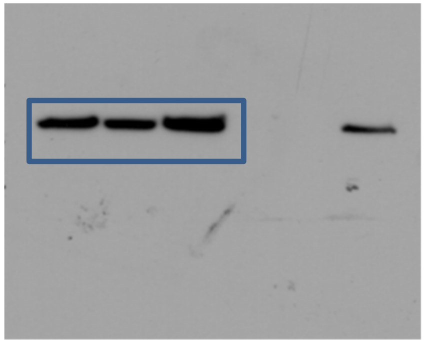

Figure 6D mTOR

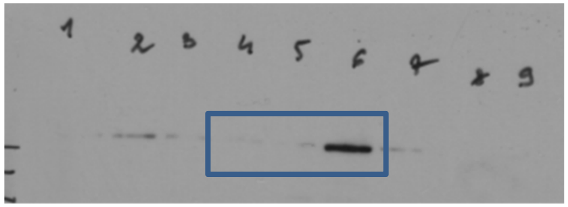

Figure 6D β-Actin

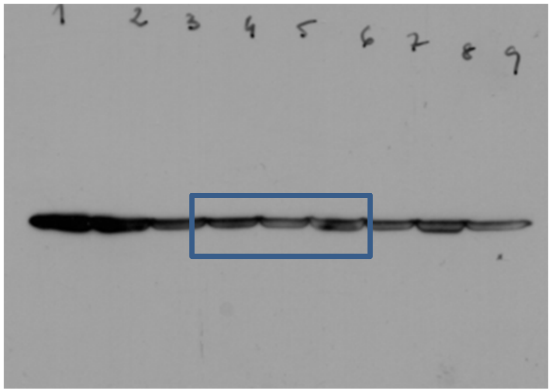

Figure 6E SLC25A1

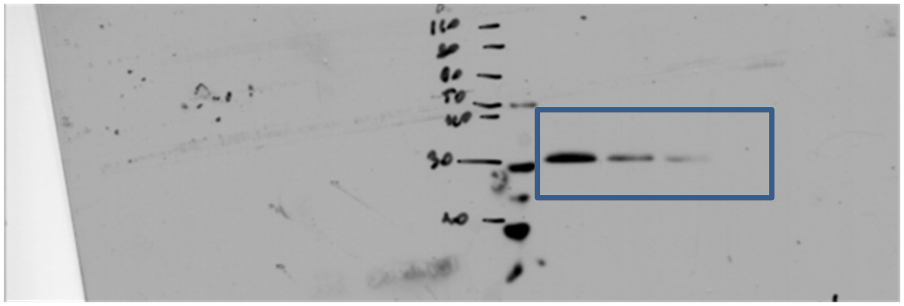

Figure 6E HSP70

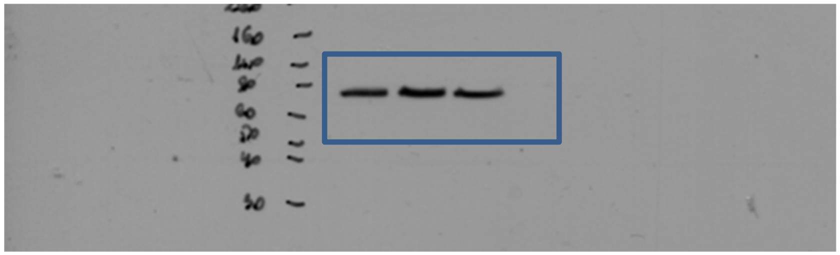

Figure 6E p53

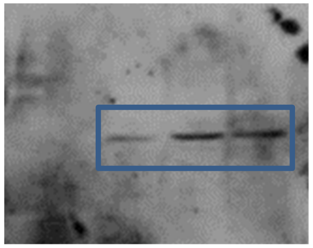

Figure 6E p21

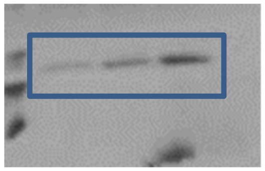

Figure 6E β-Actin

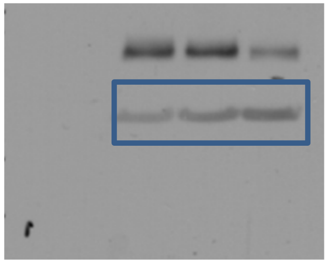

Figure 6F

mTOR

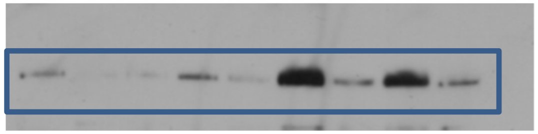

β-Actin

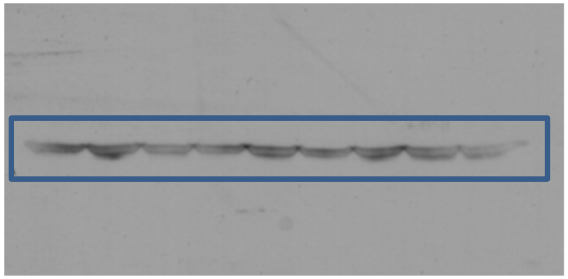

Figure 8F

SLC25A1

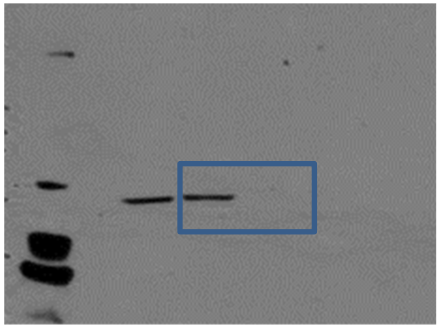

β-Actin

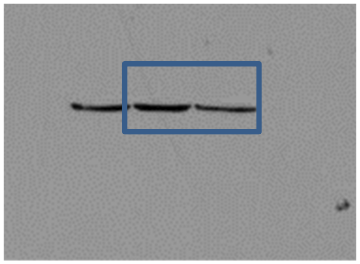

BRAIN

Figure 8I IDH1

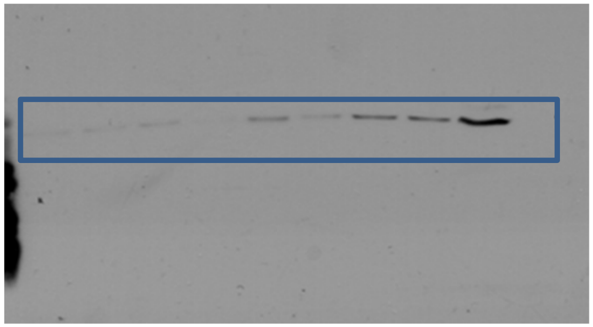

Figure 8I β-Actin

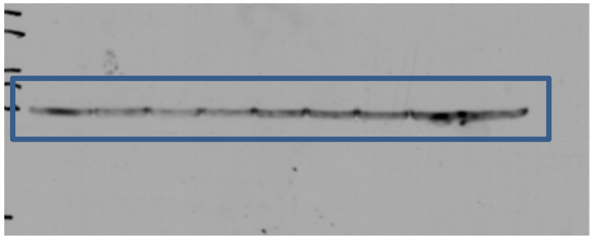

MEFs

Figure 8I IDH1

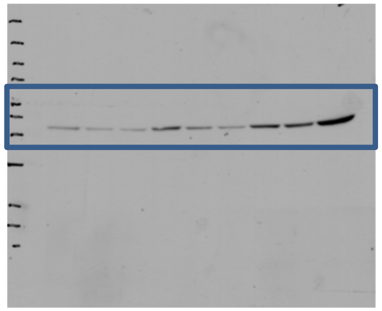

Figure 8I β-Actin

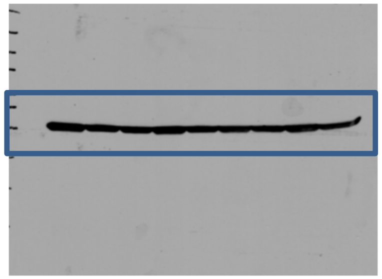

Figure 8K

p53

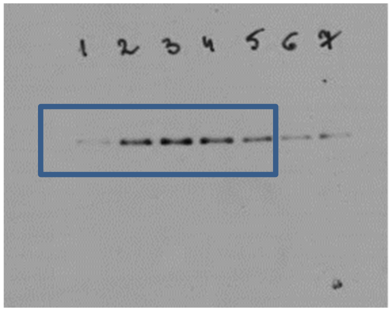

p21

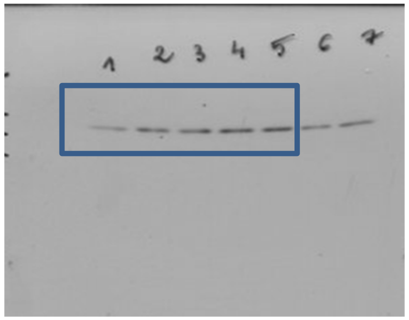

HSP70

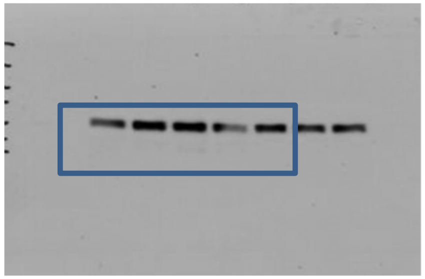

Figure 10D

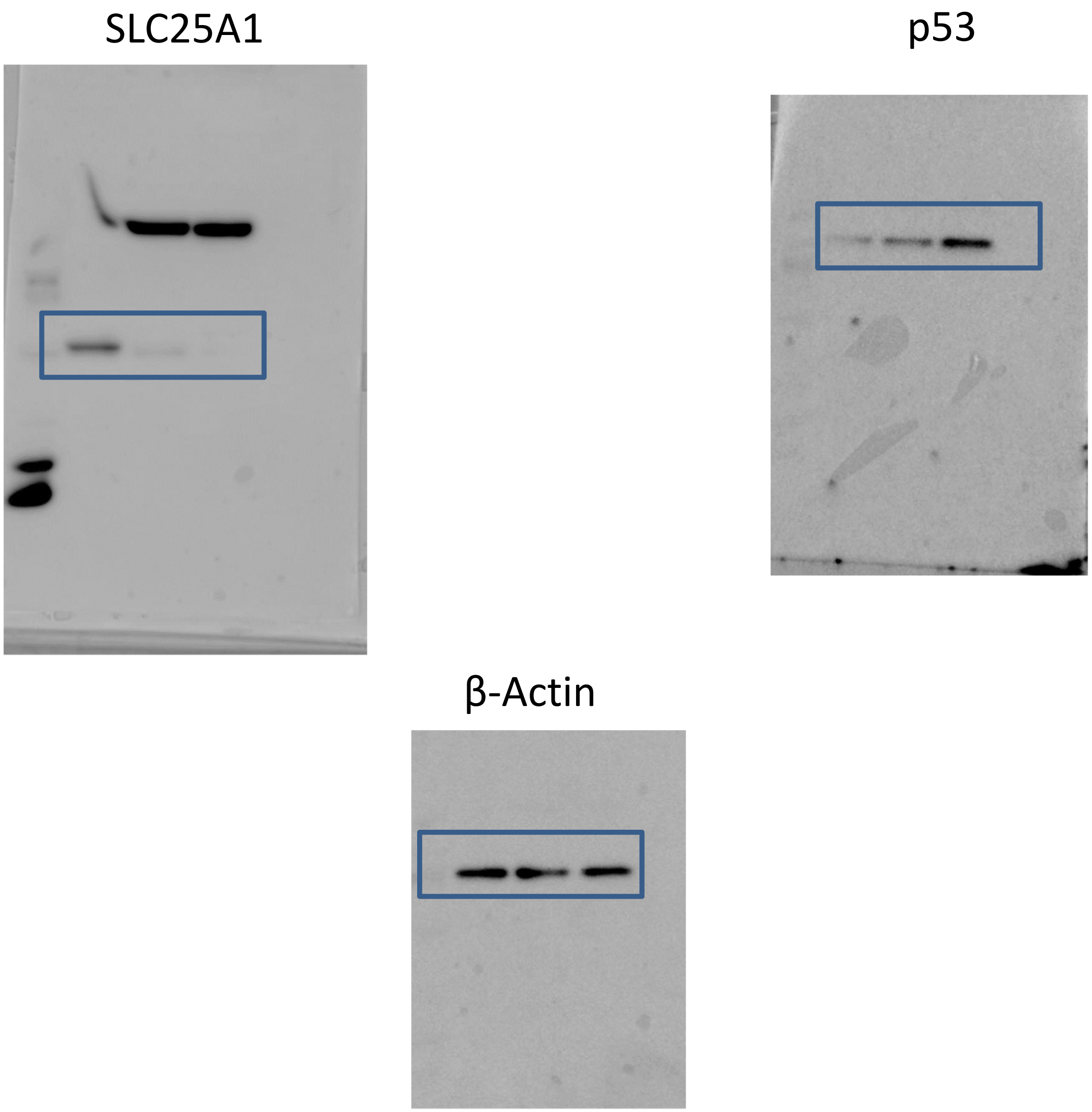

Figure 10E

p53

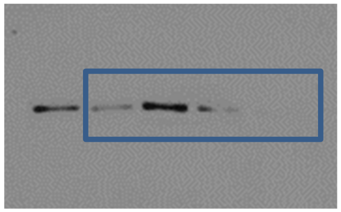

SLC25A1

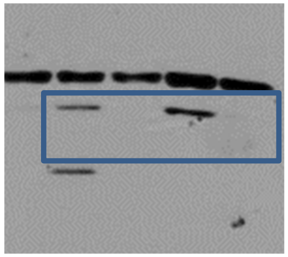

Hsp70

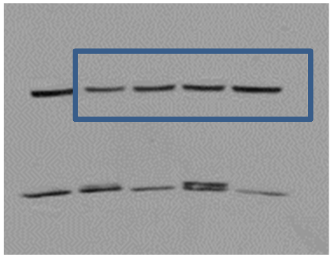

Supplementary Figure S5C

LAMIN B1

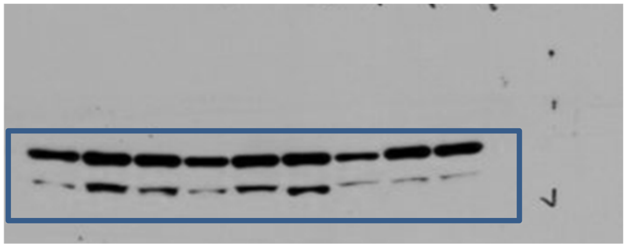

HSP70

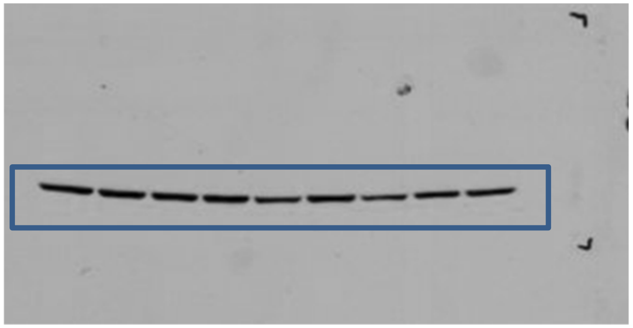

Supplementary Figure S6B

Hsp70

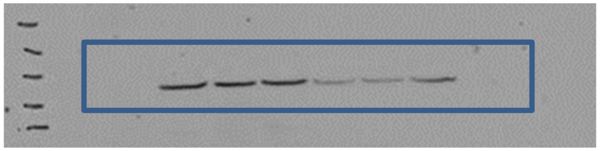

SLC25A1

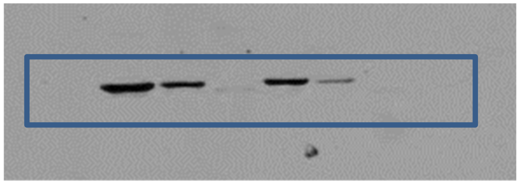

IDH2

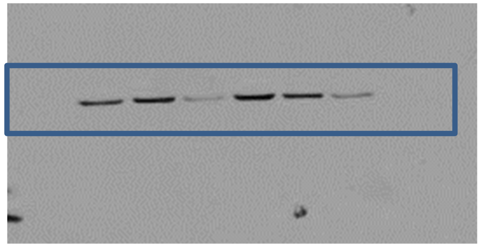

Supplementary Figure S6C

IDH2

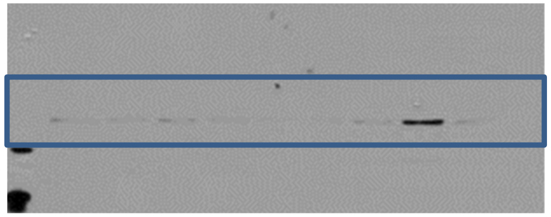

β-Actin

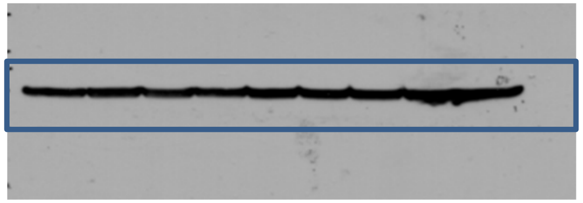

Supplementary Figure S6E

IDH1

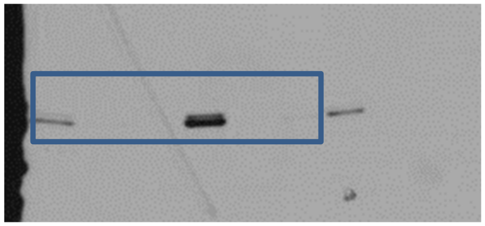

HSP70

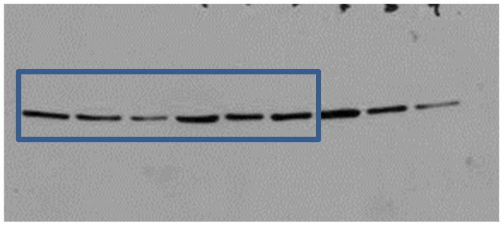

Supplementary Figure S7A

D2HGDH

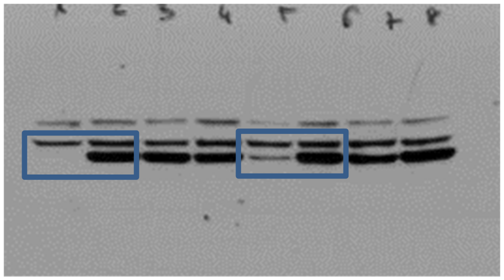

SLC25A1

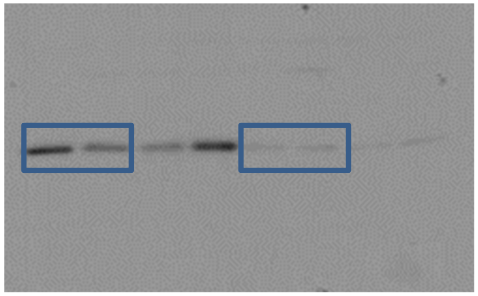

HSP70

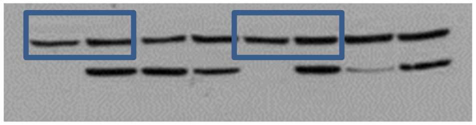

Supplement: Supplementary file 3 — Uncropped autoradiograms [file 41418_2024_1428_MOESM3_ESM.pdf]
